# Specifically horizontally tethered DNA probes on Au surfaces allow labelled and label-free DNA detection using SERS and electrochemically driven melting†
†Electronic supplementary information (ESI) available: Details of the fabrication of sphere segment void (SSV) substrates, synthesis of the modified dT monomer and DNA modifications; DNA sequences (probes and targets); p structures of synthetic modifications made to DNA; surface coverage data, data covering the range –400 mV to 1200 mV for DNA electrochemical melting and data illustrating the reduction and oxidation of methylene blue using SERS. See DOI: 10.1039/c5sc03185k


**DOI:** 10.1039/c5sc03185k

**Published:** 2015-10-08

**Authors:** E. Papadopoulou, N. Gale, J. F. Thompson, T. A. Fleming, T. Brown, P. N. Bartlett

**Affiliations:** a Chemistry , University of Southampton , Highfield , Southampton , SO17 1BJ , UK . Email: pnb@soton.ac.uk; b ATDBio Ltd , Chemistry , University of Southampton , Highfield , Southampton , SO17 1BJ , UK; c Department of Chemistry , University of Oxford , Chemistry Research Laboratory , 12 Mansfield Rd , Oxford OX1 3TA , UK

## Abstract

Controlled covalent attachment of dsDNA horizontally orientated on a gold surface is achieved through the use of a single surface-linker located approximately half way along the attached DNA probe strand. We show that horizontally oriented dsDNA on a gold surface can undergo melting and re-hybridization to target strand in solution and thus can be used for the detection of specific target DNA sequences using surface-enhanced Raman spectroscopy (SERS). We show that a range of lengths of target DNA sequences from ∼30-bases to 78-bases can be specifically hybridized to the short immobilized DNA probe sequence and adopt a horizontal orientation on the gold surface. Following thermal or electrochemically driven melting of the immobilized dsDNA, the target DNA strand diffuses away while the probe strand remains attached to the surface allowing the functionalized surfaces to be reused. The melting of the horizontally orientated immobilized dsDNA can be monitored using SERS either by employing a dye label covalently attached on the DNA target strand or by employing a binding agent selective for dsDNA. This approach of covalently immobilizing the DNA probe strand through a linker located at approximately the middle of the strand has great potential to improve the sensitivity and specificity of molecular assays that employ DNA arrays on solid surfaces.

## Abstract 2

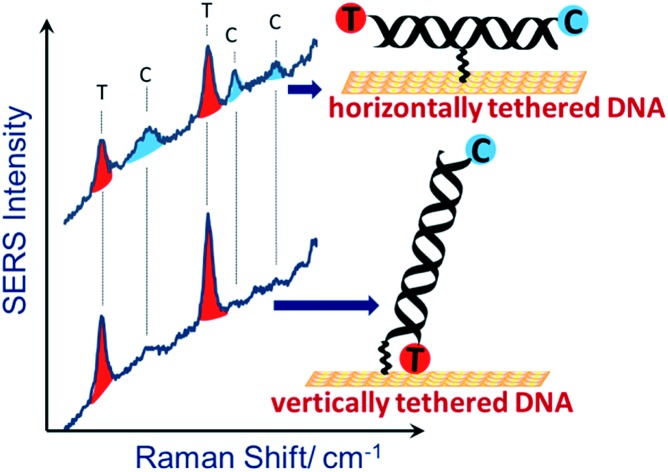
Controlled covalent attachment of dsDNA horizontally orientated on a gold surface is achieved through the use of a single surface-linker located approximately half way along the attached DNA probe strand.

## Introduction

DNA monolayers immobilised on solid surfaces have been extensively used in gene expression profiling and the detection of microorganisms using a range of optical (*e.g.* fluorescence, surface plasmon resonance (SPR),^1^ surface enhanced Raman spectroscopy^2^–^4^) and electronic sensing platforms.^5^,^6^ In a typical assay, a short single-stranded nucleic acid, the probe, is covalently immobilised on a surface *via* either the 3′ or 5′ end, through the use of reactive functional groups (*i.e.* thiols, aldehydes, epoxides, amino- and carboxyl groups). Immobilization *via* the 3′ or 5′ end allows vertical orientation of the DNA sequence on the surface.

Both short (30-base) and long (76–78 base) subsequence hybridization of the probe to a labelled complementary strand is used for the detection of a specific unknown sequence or sequences. Complex DNA mixtures are analysed with microarray technology where microscopic spots containing identical single stranded DNA (ssDNA) chains attached through the 3′ or 5′ end to the solid substrate.^7^–^9^ The microarray is then immersed in a solution containing labelled ssDNA chains whose sequences are unknown. Hybridization on a specific spot, monitored by the strength of the label signal at the position of the spot, indicates the presence of a specific sequence. This method provides high specificity and sensitivity due to the mutual selectivity between complementary strands of DNA sequences.

Even though it is often presumed that end tethered DNA sequences adopt a vertical orientation at the surface, a number of studies have shown that a variety of triggers can influence the DNA conformation and tilting angles on the surface. These triggers include surface potential,^10^,^11^ temperature, pH, ionic strength,^11^ the length of the DNA and the nature of the DNA-end that is tethered.^12^ In addition, when using ssDNA modified at one end with a surface linker care must be taken to control the surface coverage of immobilised DNA since high surface coverages lead to slow hybridization kinetics and low hybridization efficiencies due to steric effects.^13^ This problem becomes more intense when real DNA fragments are used as targets since these are generally longer (usually >80 base pairs). Optimization of the probe density^14^ and the use of hybridization rate accelerators (*e.g.* dextran sulphate,^15^ CTAB^16^) have been employed to improve the hybridization efficiencies.

An alternative methodology to the use of end tethered DNA probes, would be to specifically immobilize the probes horizontally on the surface. This orientation will automatically lead to lower probe density which can increase the hybridization efficiency as well as locating the DNA backbone closer to the sensor surface thereby allowing increased sensitivity. For example, DNA sequences that were aligned horizontally to the surface have been shown to be effective for sensitive electronic^17^ and SERS^18^–^21^ label free detection of DNA. However, in these reports the DNA was physically adsorbed on the surfaces, whereas specific attachment *via* a linker would be more efficient and more controlled for the design of molecular assays. A recent paper by De *et al.* described the specific horizontal covalent immobilization of a 15 base PNA probe on a silicon dioxide surface *via* three linker molecules attached at three locations (*γ* points) along the PNA backbone.^22^ This approach, although promising, used linkers at both ends of the probe, leaving open the possibility for the DNA to bind on the surface *via* only one end.

Herein, we report a simple, straightforward methodology for the specific covalent attachment of DNA probes using a single linker, by placing the linker approximately in the middle of the probe strand we ensure horizontal orientation of the attached dsDNA due to the rigidity of the hybridized double stranded DNA. The persistence length of dsDNA depends on the sequence and on the electrolyte composition but is generally of the order of 50 nm.^23^ Thus a dsDNA strand of around 30 bp (∼10 nm) is expected to behave as a rigid rod. The thiol linker is attached to a thymine base. The resulting covalently immobilized DNA probes can be specifically hybridized to a complementary DNA target sequence, more importantly we show that a range of target sequences of different length can be used. Target sequences can be specifically hybridized to the target DNA probes immobilized by a single, centrally placed linker.

In earlier work we used DNA conventionally attached through one end of the probe strand on Au sphere segment void (SSV) surfaces selected to give large SERS enhancements^24^,^25^ to develop assays for sensitive DNA discrimination by targeting the detection of single nucleotide polymorphism^26^,^27^ and tandem repeats.^28^,^29^ Specifically, a negative potential is applied on the Au SSV surface to electrochemically “melt” the immobilized dsDNA and this electrochemically driven melting (E-melting) is monitored by recording the SERS signal of the labelled DNA target as a function of applied potential. When the DNA target diffuses away from the surface after dsDNA dissociation, the signal of the SERS label decreases significantly. Importantly, we also showed that it is possible to monitor the electrochemically driven melting of the duplexes on the Au SSV surface utilizing the SERS signal of a binding agent (methylene blue) that can specifically interact with the double helix instead of using a label covalently attached to the target DNA.^30^ The use of a binding agent allows label free DNA analysis since the DNA target sequence can be used without any prior synthetic modification.

Here we apply the same E-melting methodology, using DNA probe strands covalently immobilized by a single linker near the middle of the strand, utilizing both a covalently attached dye and methylene blue, as a binding agent specific for dsDNA, to monitor the DNA melting. We show that the SERS spectrum of the binding agent is different for dsDNA covalently immobilized through the middle of the probe strand compared to that conventionally immobilized through one end of the probe strand, consistent with a difference in orientation of the strands, and hence the dye, in the two cases.

## Experimental

All reagents used were analytical grade and obtained from Sigma-Aldrich, unless stated otherwise.

### Oligonucleotide synthesis

Oligonucleotides were synthesized by ATDBio Ltd (Southampton, UK). Experimental details of the synthesis of the modified dT monomer and DNA modifications are included in the ESI.† All the DNA sequences used in this study are shown in Table S1.†

### Immobilization of DNA oligonucleotides on the substrate and DNA hybridization

Sphere segment voids (SSV) are prepared as described in the ESI.† For the horizontally tethered dsDNA two methods were employed (a) 0.5 μM of ssDNA probe with the dithiol located on the thymine base was hybridized in solution with 0.7 μM complementary DNA target in 10 mM phosphate buffer/0.05 M Na_2_SO_4_. The dsDNA was then immobilized on an Au SSV surface after overnight incubation at room temperature. The surface was then washed with 10 mM phosphate buffer/0.05 M Na_2_SO_4_ and the remaining gold surface was passivated by immersing the substrate in a 10 mM mercaptohexanol diluted 10 mM phosphate buffer/0.05 M Na_2_SO_4_ for 30 min. (b) 0.5 μM of ssDNA with the dithiol located on the thymine base diluted in 10 mM phosphate buffer/0.05 M Na_2_SO_4_ was immobilized on the Au SSV surface after 6 h incubation at 40 °C. The surface was rinsed thoroughly and the remaining gold surface was passivated by immersing the substrate in a 10 mM mercaptohexanol diluted in 10 mM phosphate buffer/0.05 M Na_2_SO_4_ for 30 min. After passivation with mercaptohexanol the surface was rinsed thoroughly and was immersed in 1 μM complementary target DNA solution diluted in 10 mM Tris buffer/1 M NaCl for two hours at room temperature to hybridize.

For the vertically tethered dsDNA the following procedure was employed: 1 μM of ssDNA probe with the dithiol on either the 3′ or the 5′ end diluted in 10 mM Tris buffer/1 M NaCl was immobilised on an Au SSV surface after overnight incubation at room temperature. The surface was then rinsed thoroughly and the remaining gold surface was passivated by immersing the substrate in a 10 mM mercaptohexanol diluted in 10 mM phosphate buffer/1 M NaCl for 30 min. The surface was rinsed thoroughly and immersed in 2 μM complementary target DNA solution diluted in 10 mM Tris buffer/1 M NaCl for two hours at room temperature to hybridize.

### Electrochemically driven melting procedure

Electrochemically driven melting (E-melting) experiments were carried out in a custom-built spectro-electrochemical Raman cell (Ventacon Ltd.) where the SSV substrate is used as the working electrode, a platinum wire as the counter electrode, and Ag/AgCl pellet as the reference electrode. In a typical electrochemical melting experiment, the potential was swept at 0.7 mV s^–1^ from a starting potential of –0.4 to –1.2 V in 10 mM Tris/1 M NaCl buffer. All electrochemical measurements were carried out using an EcoChemie AutolabIII potentiostat/galvanostat at room temperature. Raman spectra were acquired using a Renishaw 2000 microscope instrument equipped with a 632.8 nm He–Ne laser. The diameter of the laser spot was 1 μm. Typically, the laser power was 2.3 mW and spectra were recorded with an exposure time of 30 s.

### Data analysis

SERS spectra presented were baseline-corrected using a polynomial multipoint fitting function in Origin 9.1. The Raman intensities of the peaks are taken as height above the baseline. Origin 9.1 was used to fit sigmoidal curves to the melting profiles using the following equation
1

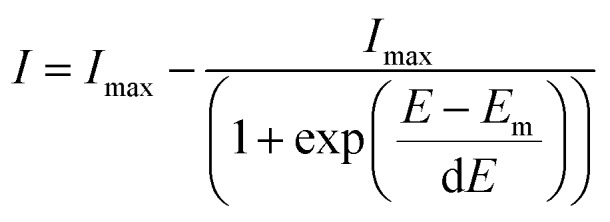

where *I* is the absolute spectral intensity of the band at 1505 cm^–1^ at the applied potential *E*, *I*_max_ is the average intensity value at the plateau for the sigmoidal curve, *E*_m_ is the melting potential when *I* equals *I*_max_/2, and d*E* is a constant that describes the sharpness of the melting curve (the gradient of the curve at *E*_m_ is *I*_max_/4d*E*).

## Results and discussion

### Horizontally tethered DNA

**Fig. 1:**
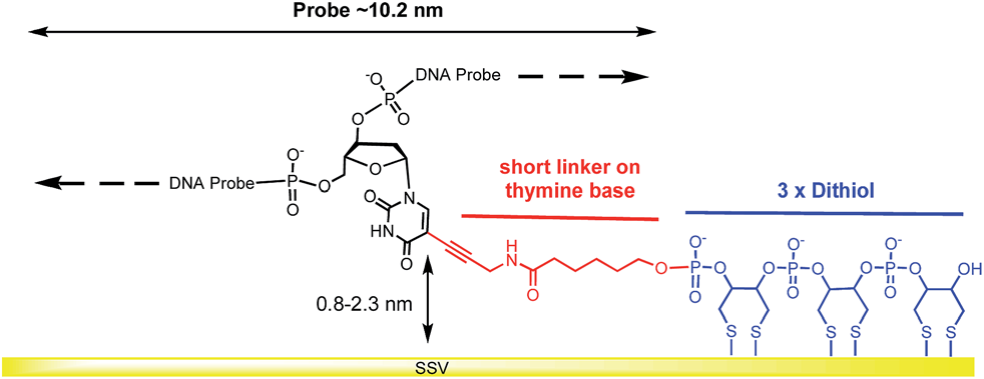
Structure of the dithiol linker attached on the thymine base.

A modified 30 base oligonucleotide probe, as shown in Table 1 (probe-1), was synthesized. The T* indicates a deoxythymidine (dT) modified with a linker consisting of three dithiols as a surface anchor and a propagylamidopentanol linker attached at the C5 position of the thymine, as shown in Fig. 1 (synthesis details are shown in the ESI†). Three dithols were used to suppress desorption of the probe at negative potentials. The modified dT was the 16^th^ base along the probe starting from the 5′ end. The length of the DNA probe is ∼10.2 nm and the length of the linker between the thymine and the Au surface (from propagylamidopentanol, phosphate and the first dithiol) can range from 0.8 nm to 2.3 nm depending on whether the alkyl chain is coiled or extended. No spacers have been used between the three dithiol units and thymine base to minimize the bending of the DNA duplex and facilitate a fixed orientation on the surface. Immobilization of the DNA probe at room temperature prior to hybridization was avoided since the DNA might coil up on the surface in an unsuitable manner. Instead, two different methods were used: (i) the DNA probe was hybridized to its target in solution to form the desired rigid duplex. The Au SSV surface was then incubated in the 0.5 μΜ dsDNA solution at room temperature, overnight, following passivation with mercaptohexanol to prevent the non-specific adsorption of DNA at the gold surface.^31^ (ii) The DNA probe was first immobilized on the surface at 40 °C for 6 h in order to allow the DNA to bind in its uncoiled form.^11^ The Au surface was then passivated with mercaptohexanol before hybridizing to its target DNA at room temperature for 2 h. It should be noted that in both cases the DNA was immobilized at low ionic strength (0.05 M Na_2_SO_4_) since there was no need to screen the duplex charge in these inherently low probe density surfaces.

**Table 1:** DNA probe sequences used in this study^*a*^

Probes	Sequence (5′-3′)
Probe-1 (P1)	KATATCATCTTTGGTGT*TTCCTCATGCTTTA
5′-End thiol P1	SSSHH-ATATCATCTTTGGTGTTTCCTCATGCTTTA
3′-End thiol P1	ATATCATCTTTGGTGTTTCCTCATGCTTTA-HHSSS
Probe-2 (P2)	KCACTGACAGTCAGTT*TGTGGTAGGATGCT

^*a*^K = hexynol, T* = monomer 1-dithiol-dithiol-dithiol, S = dithiol monomer, H = hexaethylene glycol.

Two further versions of probe-1 were prepared (Table 1) to compare the SER spectra for the DNA immobilized in three different ways: by covalent attachment at the 5′ end, at the 3′ end and through a single linker in the middle of the probe strand. These three versions of probe-1 were hybridized to a fully complementary strand labelled with Texas Red® at the 5′ end and Cy3B at the 3′ end (see Table S1†).

**Fig. 2:**
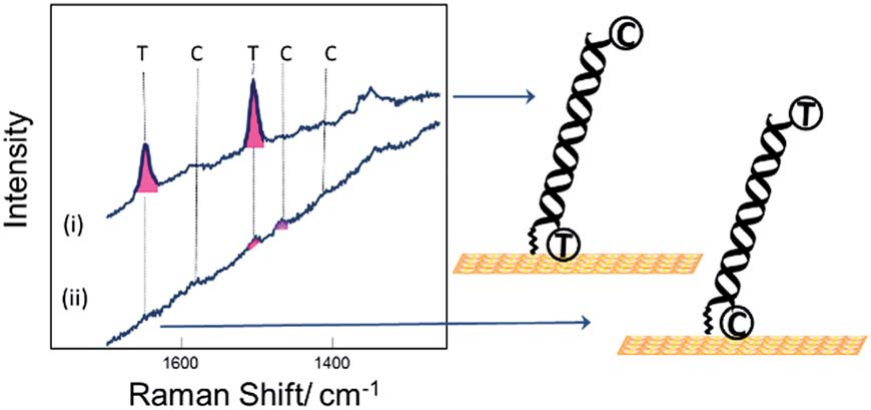
SER spectra for DNA probe-1 with the dithiol at the (i) 3′ end and (ii) 5′ end, hybridized to a complementary DNA target. The DNA target was labelled with Texas Red® (T) and Cy3B (C) at the 5′ and 3′ ends respectively.

Fig. 2 shows SER spectra for the dsDNA immobilized in the conventional way through covalent attachment at either the 5′ or 3′ ends. In both cases the gold surface has been modified with mercaptohexanol after immobilization of the probe strand. The use of mercaptohexanol is known to suppress adsorption of DNA on the bare gold surface and to encourage the dsDNA to adopt a vertical orientation on the surface.^31^

The spectra for the two different probe orientations show significant differences. In Fig. 2(i) where the Texas Red® is close to the surface and Cy3B is ∼10 nm from the surface, the Texas Red® bands dominate the spectrum and the Cy3B bands are not visible; whereas in Fig. 2(ii), when Texas Red® is positioned at the opposite end from the attachment point (∼10 nm from the surface), the fluorescence is not quenched and the SERS bands are almost completely masked.

**Fig. 3:**
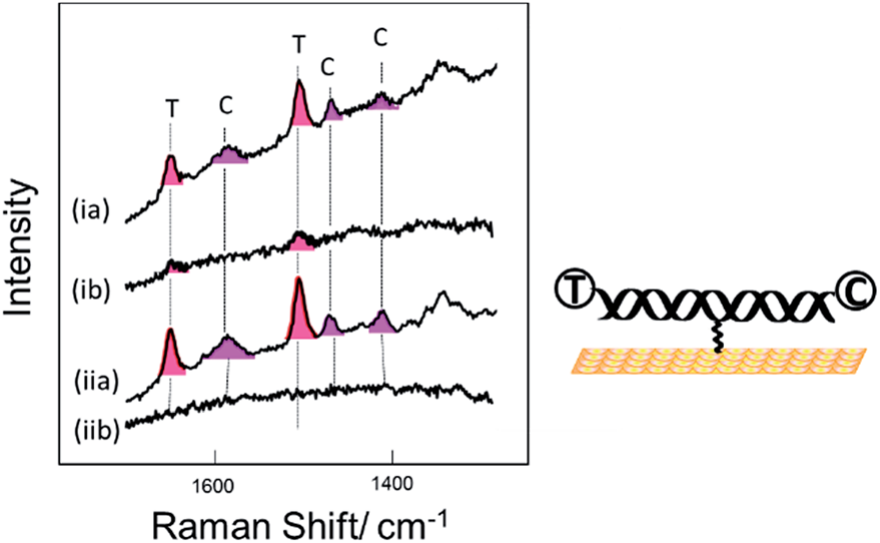
SER spectra for DNA probe-1 attached to the surface through the thiol linker attached to the thymine base near the middle of the probe strand. Two sets of data are shown for the probe immobilized on the surface by (i) hybridization to its target in solution prior to immobilization to the surface and (ii) by immobilization of the single strand probe at 40 °C followed by hybridization of the surface bound probe to the target. In both cases spectra are shown for hybridization of the probe to (a) the fully complementary target strand and (b) to a non-complementary target stand. The DNA target strands were labelled with Texas Red® (T) and Cy3B (C) at the 5′ and 3′ ends respectively.

Fig. 3 shows SER spectra for the same probe sequence attached to the surface through the single linker on the middle of the probe strand. In this case the probe was attached to the surface following two different protocols. For Fig. 3(i) the probe was first hybridized to the target in solution and then the hybridized probe was attached to the gold surface followed by passivation of the gold surface with mercaptohexanol. For Fig. 3(ii) the single stranded probe was immobilized on the surface at 40 °C, conditions chosen to avoid the probe coiling up,^11^ the Au surface was then passivated with mercaptohexanol, and then the immobilized probe was hybridized to the target DNA (see Experimental section for full details). In both cases results are shown for hybridization to (a) the fully complementary target and (b) to a non-complementary target. The fully complementary target strands were labelled on the 5′ end with Texas Red® and on the 3′ end with Cy3B (see Table S1† for details) while the non-complementary strand was only labelled on the 5′ end with Texas Red.

Comparing the spectra in Fig. 3 for the labelled complementary (a) and non-complementary (b) targets, we can see that the probe is highly selective for the complementary sequence as expected. In Fig. 3(ib) there is a small signal for Texas Red®. We attribute this to some residual non-specific adsorption of the labelled non-complementary target onto the gold surface that was not displaced by the subsequent treatment with mercaptohexanol. This is not seen in Fig. 3(iib) when the surface is only exposed to the non-complementary target after passivation with mercaptohexanol.

Comparing the spectra in Fig. 3(ia) and (iia) we can see that when the probe is hybridized to the labelled complementary target strand, either before or after attachment of the probe to the surface, there is no significant difference in the spectra. This result clearly shows that the labelled target can still hybridize to the probe even when it is immobilized on the surface by a linker in the centre of the probe strand. The spectra show clear bands corresponding to both the Texas Red® and the Cy3B labels. It is notable that even though the two dyes are present at exactly the same surface concentration, the Texas Red® bands are stronger than those of Cy3B. There are several factors that may contribute to this. First, different linkers were used to attach each dye to the target DNA (see Fig. S1†), and it is possible that Texas Red® adopts a more favoured orientation on the surface compared to Cy3B. Second, Texas Red® has a stronger resonance contribution (*λ*_max_ 589 nm) with the 633 nm laser used here than the Cy3B dye (*λ*_max_ 558 nm). A similar difference in SERS intensity has been observed when two different DNA strands individually labelled with the two dyes were immobilized vertically on the surface with the dyes in a similar location.

Comparing the spectra in Fig. 3 for the centrally bound probe with those in Fig. 2 for the two end-bound probes it is clear that for the centrally bound probe bands for both dyes are seen together. The presence of bands associated with both dye labels is consistent with the horizontal orientation of the dsDNA on the Au surface placing both dye labels close to the surface so both experience significant surface enhancement and the fluorescence from both is heavily quenched.

### Melting and rehybridization

**Fig. 4:**
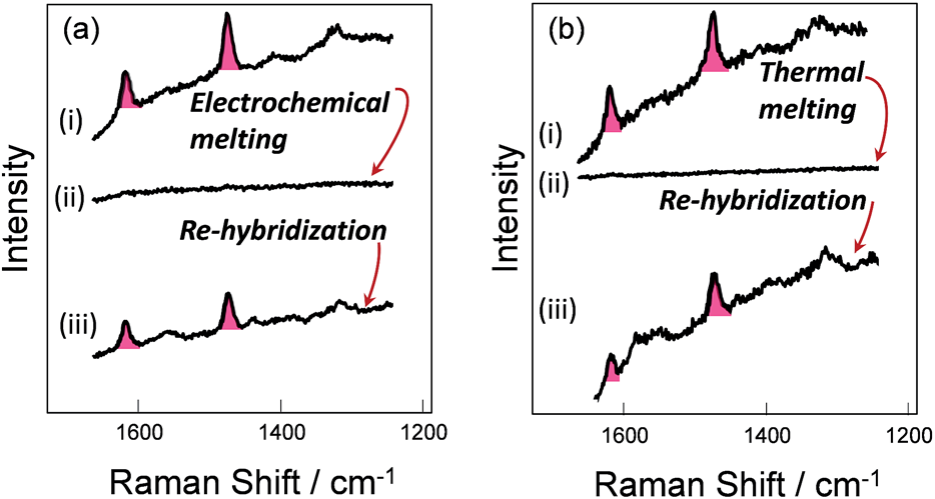
(a)-(i) and (b)-(i), SER spectra of DNA probe-1 (the dithiol is attached to the probe on the thymine base) hybridized to the fully-complementary target labelled with Texas Red®. (a)-(ii) After electrochemical melting at –1.2 V and (b)-(ii) after thermal melting at 80 °C, in either case, the SERS signal is lost due to DNA denaturation, but recovered (a)-(iii), (b)-(iii) after re-hybridization at room temperature with the labelled target.

To verify the robustness and practicality of the use of the thiol linker attached on the thymine base, the horizontally tethered dsDNA was denatured and then re-hybridized. For these experiments the target DNA was labelled with a single Texas Red® fluorophore at the 5′ end. After DNA denaturation, either electrochemically (by application of –1.2 V *vs.* Ag/AgCl), or thermally (by heating to 80 °C), the Texas Red® SERS signal was lost, Fig. 4(a)-(ii) and (b)-(ii), due to the labelled DNA target being released and diffusing away from the surface. In both cases, the SERS signal was recovered when the immobilized probes were re-hybridized, Fig. 4(a)-(iii) and (b)-(iii), clearly demonstrating that the probe horizontally oriented through the thiol linker attached to the thymine remained on the surface during denaturation by either method and that it could undergo re-hybridization so that the functionalized substrates could be reused. The slight reduction in the SERS signals after electrochemical denaturation, compare Fig. 4(a)-(i) and (b)-(i), probably reflects some reductive electrochemical desorption of the thiol linked DNA probe from the Au surface at –1.2 V *vs.* Ag/AgCl.

### Surface coverage

The surface density of DNA probes at a gold electrode was estimated using the chronoamperometric method of Steel *et al.*^32^ Previously we found the surface coverage of dsDNA immobilized through the three dithiol linker at the 3′ or 5′ end to be ∼1.6 × 10^12^ molecules per cm^2^ (on average the DNA molecules are 8.5 nm apart). We found the surface coverage of the dsDNA immobilized horizontally through the thiol linker attached on the thymine base to be 4.2 × 10^11^ per cm^2^ (on average of the DNA molecules are 15 nm apart), demonstrating the lower density of the DNA on the surface using this new approach (see Fig. S2†). For this experiment, the duplex was hybridized in solution and then the duplex strand (0.5 μM of dsDNA in 0.05 M Na_2_SO_4_) was immobilized on the SSV Au surface overnight.

### Longer target strands

**Fig. 5:**
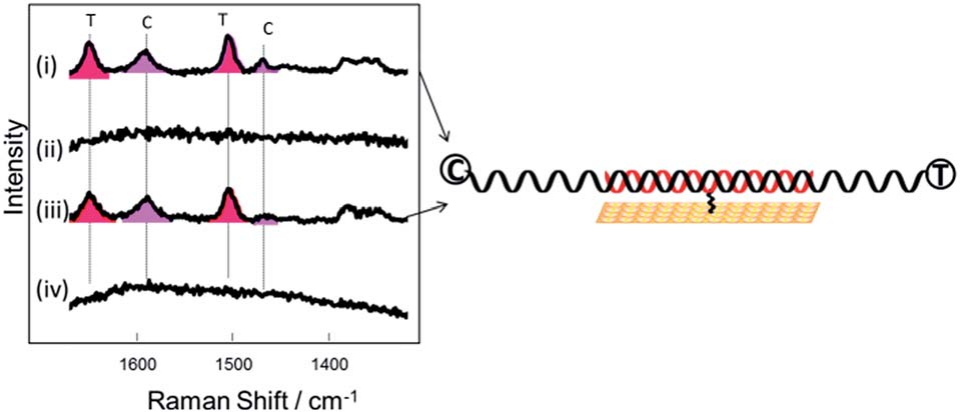
SER spectra of (i and ii) DNA probe-1 after hybridization to (i) a 78-base complementary target sequence (target 3) (ii) a 79 base non complementary sequence (target 5) (iii and iv) DNA probe-2 after hybridization to (iii) a 77-base complementary target sequence (target 4) (iv) a 79 base non complementary sequence (target 5).

For the experiments described above, the DNA target sequences had the same length as the probes (30 bases). To assess the functionality of the current methodology for bio-diagnostic applications, we carried out experiments utilizing longer (76–79 base) target sequences. This more closely resembles the conditions required in diagnostics assays where PCR amplification is used and where the PCR products are normally longer compared to the probe sequences. Two different probes were used (Table 1), the 30-base DNA probe-1 as described above and a 29-base DNA probe-2. Probe-2 was also modified with a similar three dithiol linker as probe-1 through a modified dT which was the 15^th^ base along the probe starting from the 5′ end. After immobilization of each probe on a gold SSV surface at 40 °C, the probes were hybridized with both complementary and a non-complementary DNA target sequences during separate experiments. The length of the DNA target sequences was in the range of 76–79 base (Table S1†). Each complementary target sequence was designed to contain a section that was fully complementary to probe 1 (30 bases) and probe 2 (29 bases) respectively. Upon hybridization, substantial overhanging sequences were present at both ends, for both of the probes. The complementary DNA targets were labelled on each end with different fluorophores, Texas Red® at the 3′-end and Cy3 at the 5′-end. The non-complementary target was labelled with a Texas Red® fluorophore at the 3′-end. As previously, the surface was passivated with mercaptohexanol before hybridization to the target DNA. Fig. 5 shows the SER spectra of the DNA probes with the complementary and non-complementary target. Bands for both Texas Red® and Cy3 are visible when the DNA probes were hybridized to the complementary long DNA target, whereas no bands of Texas Red® are observed with the non-complementary sample. These results clearly show that the horizontally orientated DNA probes can be used with longer target strands such as those generated by PCR amplification of an analyte sequence.

### Electrochemically driven melting experiments

**Fig. 6:**
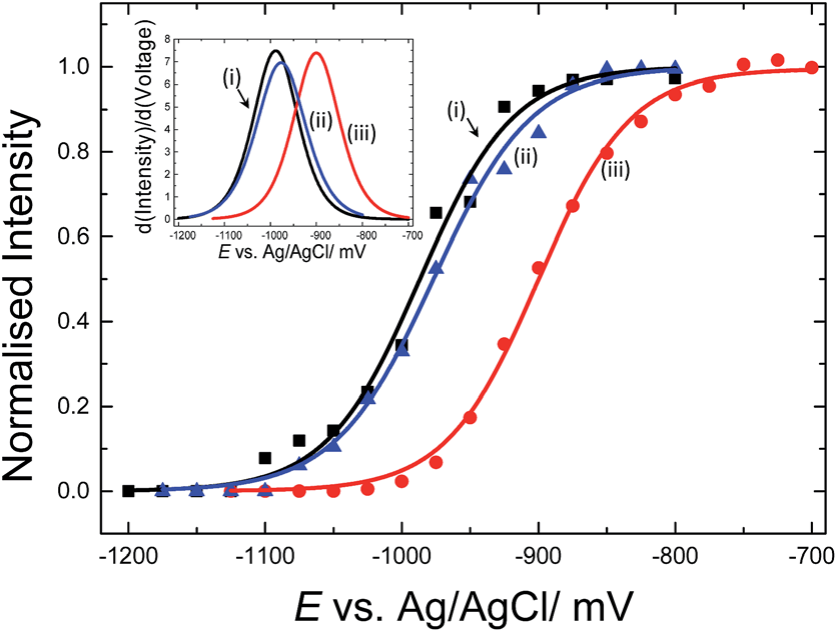
Electrochemically driven melting curves for (i and ii) horizontally tethered dsDNA after (i) hybridization in solution followed immobilization of the duplex strand (0.5 μM dsDNA in 0.05 M Na_2_SO_4_) on an Au SSV substrate, (ii) immobilization of ssDNA probe (0.5 μM ssDNA in 0.05 M Na_2_SO_4_) at 40 °C on an Au SSV substrate followed by hybridization on the surface and (iii) vertically tethered dsDNA on an Au SSV substrate. Each data set was fitted to eqn (1). The potential was swept at a scan rate of 1 mV s^–1^ in 10 mM Tris buffer (pH 7.2) containing 1 M NaCl.

E-melting experiments monitored by the intensity of the Texas Red® band at 1505 cm^–1^, were performed using both horizontally and vertically tethered dsDNA. For those experiments probe-1 hybridized to its complementary short DNA target was utilized. Briefly, in each case the potential was ramped from a starting potential of –400 mV to a final potential of –1200 mV *vs.* Ag/AgCl. SERS spectra were recorded at 25 mV intervals. As the DNA duplex melts, the Texas Red® labelled probe diffuses away from the surface and the Texas Red® signal is decreased. Fig. 6 shows the E-melting curves for both horizontally tethered (using both immobilization methods) and vertically tethered DNA, constructed by plotting the intensity of the Texas Red® band at 1505 cm^–1^ against the applied potential (the data over the range –400 mV to –1200 mV are shown in Fig. S3†). The data were fitted to the eqn (1) in order to plot the E-melting curve.

It is evident that horizontally tethered DNA can be electrochemically melted on the gold surface. In addition, the electrochemically driven melting profiles are very similar for horizontally tethered DNA assembled on the surface under either set of conditions and clearly distinguishable from the vertically tethered DNA melting profile. There is a significant shift in the melting potential of ∼80 mV when the horizontally tethered DNA is used. Even though the DNA backbone in the horizontal orientation is closer to the sensor surface a more negative potential is required to denature the dsDNA as compare to that of the similar vertically tethered DNA. Given our previous experiments on PNA–PNA melting where we showed that the DNA melting is not simply due to electrostatic repulsion of the sugar–phosphate backbone away from the surface,^33^ this result is not implausible.

### Label-free detection of dsDNA utilizing horizontal configuration

**Fig. 7:**
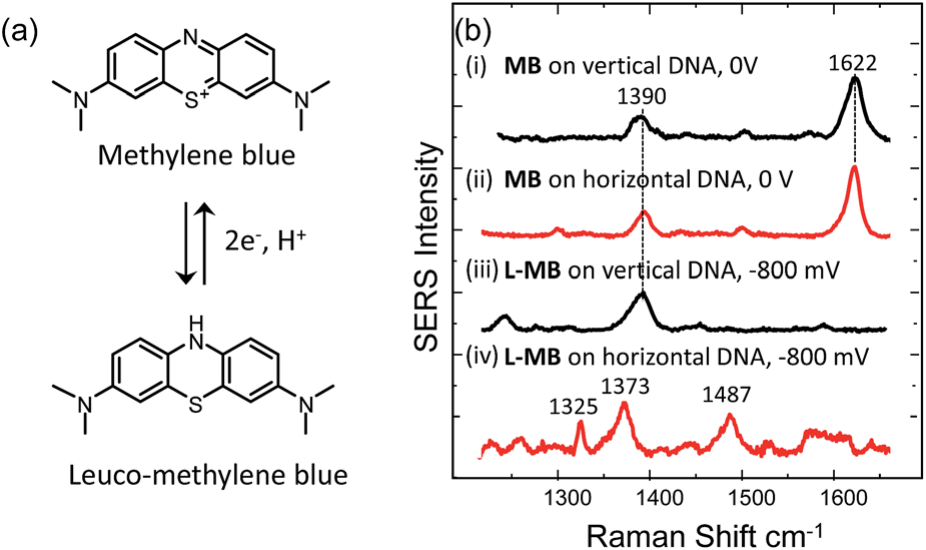
(a) Structures of methylene blue and leucomethylene blue produced after electrochemical reduction. (b) SERS spectra of methylene blue bound to vertically oriented (i)–(iii) surface tethered dsDNA at (i) 0 V and (ii) –800 mV and to horizontally oriented surface tethered dsDNA at (ii) 0 V and (iv) –800 mV. All the potentials are *vs.* Ag/AgCl.

We have also tested the ability of the system to detect DNA hybridization as well as to monitor DNA melting with the use of a binding agent selective for dsDNA. Methylene blue has been used previously with vertically aligned dsDNA tethered to the surface through the 5′-end to monitor the electrochemically driven melting and discriminate DNA sequences in the CFTR gene.^30^ Given that the DNA backbone is closer to the surface when the DNA is horizontally tethered, better sensitivity is anticipated. After the DNA duplex was immobilized horizontally on the surface, the SSV substrate was immersed to a 0.1 mM solution of methylene blue (0.1 M phosphate buffer/0.05 M Na_2_SO_4_) for 2 h. The substrate was then rinsed with buffer to remove any weakly interacting or excess binding agent. Fig. 7(b) shows the SER spectrum of methylene blue (at 0 V) and leuco-methylene blue (at –800 mV) bound on horizontally tethered dsDNA. The corresponding SER spectra on vertically tethered DNA are included for comparison.

The spectrum appears to be identical to that recorded when methylene blue was bound on vertically tethered DNA.^30^ As previously reported, methylene blue is reduced at negative potentials to its colourless leuco form, which is not in resonance with the 633 nm excitation wavelength used here. Interestingly, the SER spectrum of leuco-methylene blue was found to be different from that recorded on vertically tethered DNA (Fig. 7(b)). This can be explained by considering the orientation of the methylene blue with respect to the gold surface. The binding mechanism of the leuco form occurs mainly *via* a minor–groove interaction.^34^ Therefore, in the vertical DNA orientation methylene blue is expected to be orientated nearly perpendicular to the gold surface. In our new design, methylene blue is expected to have a near-horizontal orientation to the gold surface. On the basis of surface selection rules, vibrations that gain their Raman intensity from a change in polarizability tensor perpendicular to the surface are expected to be favourably enhanced.^35^,^36^ Hence, differences in the orientation of a molecule on the metal surface would be expected to perturb the relative enhancement of its modes. With the oxidized form of methylene blue the spectra were found to be the same as with our previous results. This difference can be attributed to the additional enhancement the molecule gains when it is in resonance with the excitation wavelength. These observations show that it is possible to deduce useful information on the intercalation mechanism of binding agents/drugs that interact with DNA by utilizing dsDNA immobilized in both orientations.

**Fig. 8:**
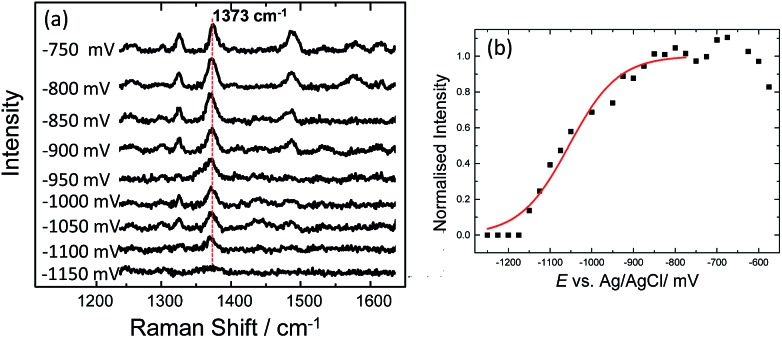
(a) SER spectra of leuco-methylene blue bound to horizontally tethered dsDNA. The spectra were recorded at different applied potentials *vs.* Ag/AgCl as shown in the figure. The potential was swept at a scan rate of 0.7 mV s^–1^ in 10 mM Tris buffer (pH 7.2) containing 1 M NaCl. (b) Plot of changes in the absolute signal intensities of the band 1373 cm^–1^ as a function of applied potential. The red line shows the fitted E-melting curve to eqn (1). The results clearly show that horizontally tethered DNA targets can be detected and analysed using a label-free electrochemically driven melting experiment.

Using SERS it was also possible to monitor the reduction and oxidation on methylene blue bound on horizontally tethered DNA (see Fig. S4†). As the methylene blue gradually reduces, the absolute signal intensities of the bands at 1622 and 1388 cm^–1^ corresponding to the aromatic C=C and C=N ring stretches fall significantly and eventually disappear at ∼–500 mV. Upon reversing the potential, the bands at 1622 and 1388 cm^–1^ reappear and restore their intensity as the molecule is oxidized and is once again in resonance with the excitation wavelength (Fig. S4†). The full recovery of the methylene blue SERS spectrum on the reverse scan demonstrates that the leuco-methylene blue remains associated with the dsDNA. Interestingly new bands start to appear at ∼–600 mV, the most prominent are the bands at 1325 cm^–1^, 1373 cm^–1^ and 1487 cm^–1^ (Fig. 8(a)). Among them, the most intense is the band at 1373 cm^–1^ which can be used to monitor the DNA melting. Upon driving the potential cathodic beyond –1100 mV *vs.* Ag/AgCl there is an irreversible loss in SERS intensity, which can be attributed to the electrochemically driven denaturation. The E-melting curve is shown in Fig. 8 along with a set of SERS spectra as a function of potential.

The melting profile of the dsDNA monitored with methylene blue appears to be broader compared to that of the dsDNA monitored with Texas Red® (Fig. 4(i) and (ii)). The melting potential was also shifted by ∼65 mV when methylene blue was used. These slight differences might be expected due to the different modes of attachment of the two dyes.

## Conclusions

This work demonstrates a completely new simple and effective methodology for the covalent attachment of DNA probes on gold surfaces that promotes horizontal orientation of the dsDNA. It is based on the use of a single surface-linker which is placed approximately in the middle of a DNA probe. The methodology is sensitive for the discrimination of complementary and non-complementary DNA, more importantly we showed that it can be applied for the detection of long (∼78 base) DNA target sequences. The DNA duplexes can also be melted on the surface and their melting profile can be monitored using either a labelled DNA target or a completely unmodified target with an added binding agent specific for double stranded DNA (*i.e.* methylene blue). Finally, we showed that combining horizontal and perpendicular DNA on surfaces, it is possible to deduce information on the orientation of the binding agent with respect to the DNA duplex and the solid surface. This information can ultimately lead to important conclusions with respect to the intercalation mechanisms of the binding agents with dsDNA.

This new immobilization strategy can be applied to a wide range of optical or electronic biosensors and has the potential to improve the overall sensitivity of DNA sensors. We believe that the same methodology can be applied to successfully induce horizontal orientation of the DNA on surfaces up to the persistence length of dsDNA (>150 bp).

## Supplementary Material

Supplementary information
