# Supplementary material for: Specifically horizontally tethered DNA probes on Au surfaces allow labelled and label-free DNA detection using SERS and electrochemically driven melting
Source: Chem Sci. 2015 Oct 8;7(1):386–93. doi: 10.1039/c5sc03185k (PMC5952265; doi:10.1039/c5sc03185k)
Supplement: Supplementary file 1 [file SC-007-C5SC03185K-s001.pdf]

Electronic Supplementary Material (ESI) for Chemical Science.
This journal is © The Royal Society of Chemistry 2015

Supplementary Information

Specifically horizontally tethered DNA probes on Au surfaces allow labelled and label-free DNA detection using SERS and electrochemically driven melting

Evanthia Papadopoulou,[†ǁ] Nittaya Gale,[‡] Jamie F. Thompson,[†] Thomas A. Fleming, [†ǁ] Tom Brown,[§] Philip N. Bartlett.*[†]

† Chemistry, University of Southampton, Highfield, Southampton, SO17 1BJ, U.K, ‡ ATDBio Ltd, Chemistry, University of Southampton, Highfield, Southampton, SO17 1BJ, UK, §Department of Chemistry, University of Oxford, Chemistry Research Laboratory, 12 Mansfield Rd, Oxford OX1 3TA, UK.

Experimental

The synthesis of modified dT monomer, 1

Propargylamine was coupled to 6-hydroxyhexanoic acid under amide coupling conditions with EDC-HCl. Levulinyl protection of the 6-hydroxy-*N*-(prop-2-yn-1-yl)hexanamide hydroxyl group was achieved with levulinic anhydride that was prepared *in-situ* from levulinic acid with carbodiimide coupling reagent, DCC. The levulinyl protected hexanamide **3** was then coupled to 5-iodo-dU **4** under Sonogashira palladium catalysed cross-coupling conditions to afford compound **5**. Finally a 3'-phosphitylation was performed with standard phosphitylating reagent to produce the modified monomer **1** in good yield.

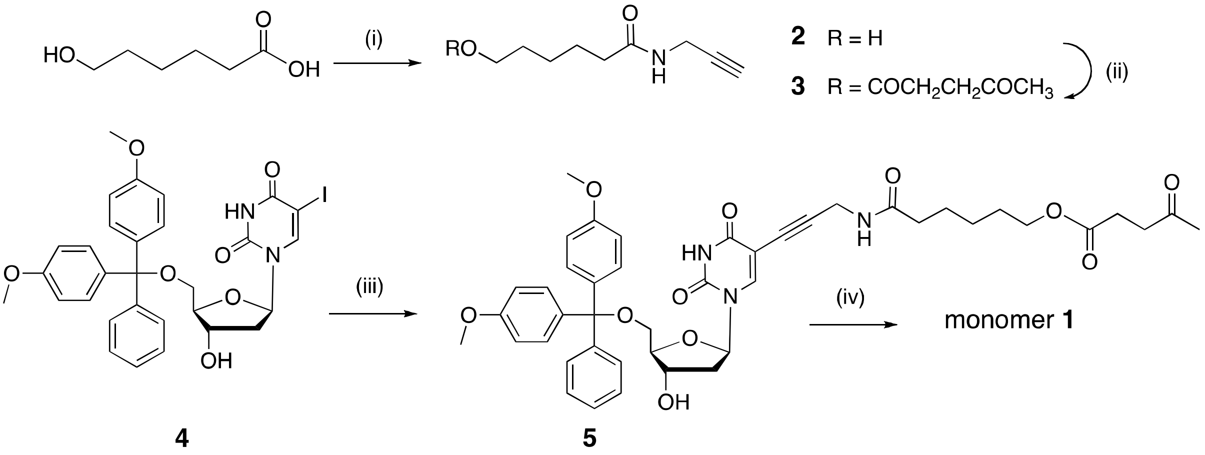

Scheme 1: Synthetic route to levulinyl-protected modified dT (i) Propargylamine (1.5 eq.), EDC-HCl (1.5 eq.), DMF, rt, 18 h, 67 %; (ii) levulinic anhydride (prepared *in-situ:* prepared from levulinic acid, DCC, Et_2O, rt, 2 h) (1.4 eq.), DCM:DMF (2:1), rt, 1.5 h, 70 %; (iii) Copper^{I} Iodide (0.25 eq.), DMF:TEA (10:1), **3**, Pd^{0}(PPh$_3$)$_4$ (0.1 eq.), rt, 1.5 h, 59 %; (iv) chloro(diisopropylamino)-β-cyanoethoxyphosphine (1.2 eq.), DIPEA (2.0 eq.), DCM, rt, 3.5 h, 74 %.

6-hydroxy-*N*-(prop-2-ynyl)hexanamide, **2**

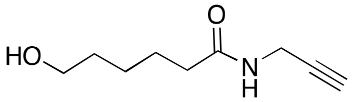

Propargylamine (0.36 g, 5.7 mmole, 1.5 eq.) was added to 6-hydroxyhexanoic acid (0.5 g, 3.8 mmole, 1.0 eq.) in DMF (10 mL). To this reaction mixture, EDC-Cl (1.09 g, 5.7 mmole, 1.5 eq.) was added and the reaction stirred under an Argon atmosphere for 18 h. Upon completion, the reaction was concentrated to dryness *in vacuo*, redissolved in DCM (10 mL) and extracted to aqueous at pH 14 through addition of NaOH (aq., 2 M). The aqueous was neutralised to pH 7 with HCl (aq., 2 M) and the product extracted with butan-1-ol. The solution was concentrated *in vacuo* to give an orange-coloured oil. This oil was diluted with DCM (10 mL) and MeOH (2 mL) and filtered to remove insoluble salts. The filtrate was concentrated *in vacuo* and purified (column chromatography, 0-5% MeOH/EtOAc) to give 6-

hydroxy-*N*-(prop-2-yn-1-yl)hexanamide as a colourless oil. (0.43 g, 2.6 mmole, 67%); **$C_9H_{15}NO_2$ M_W: 169.22**

R_f (15% MeOH/DCM + TEA) = 0.44

^{1}H NMR (300 MHz, DMSO-*d*$_6$) δ 8.20 (t, *J* = 5.4 Hz, 1H, NH), 4.33 (t, *J* = 5.1 Hz, 1H, OH), 3.83 (dd, *J* = 5.5, 2.6 Hz, 2H, $NHC\underline{H_2}$), 3.43 – 3.30 (m, 2H, $C\underline{H}_2CO$), 3.06 (t, *J* = 2.5 Hz, 1H, CH), 2.06 (m, 2H, $C\underline{H}_2OH$), 1.58–1.32 (m, 2H, $C\underline{H}_2CH_2C\underline{H}_2CH_2OH$), 1.32 – 1.13 (m, 2H, $C\underline{H}_2CH_2CH_2OH$).

^{13}C NMR (75 MHz, DMSO-*d*$_6$) δ 171.87 (C), 81.36 (C), 72.74 (CH), 60.60 (CH_2), 35.13 (CH_2), 32.27 (CH_2), 27.69 (CH_2), 25.19 (CH_2), 25.08 (CH_2).

LRMS: [ES^-MS, MeCN] m/z (%): 187.1 ($[M+18]^-$, 100 %)

HRMS: [ESI^+] m/z: found: 192.0995 $(M+Na)^+$; calcd.: 169.2218 $(M)^+$

6-oxo-6-(prop-2-ynylamino)pentyl 4-oxopentanoate, **3**

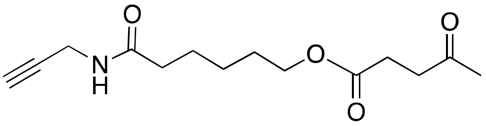

Levulinic acid (2.32 g, 2.05 mL, 20.0 mmole, 2.0 eq.) was dissolved in anhydrous Et_2O and stirred with molecular sieves (3Å) under Argon. To this solution, DDC (2.05 g, 10.0 mmole, 1.0 eq.) was added with instant observable precipitation of the dicyclohexylcarbamide by-product; the reaction was determined complete after 2 h. The reaction mixture was filtered through Celite® and washed with Et_2O then concentrated *in vacuo* to give a colourless oil that was dried under high vacuum to give levulinic anhydride as a clear/white liquid/solid (1.33 g, 12.4 mmole, 62%). The anhydride, prepared *in situ* (0.6 g, 2.8 mmole, 1.4 eq.) was dissolved in anhydrous DCM (3 mL) and anhydrous DMF (1.5 mL), cooled to 0°C. 6-hydroxy-*N*-(prop-2-yn-1-yl)hexanamide, (0.36 g, 2.0 mmole, 1.0 eq.) was dissolved in anhydrous DCM (2 mL) and anhydrous DMF (1.5 mL) and added to the stirred, cooled anhydride solution. The reaction mixture was allowed to warm to rt and proceed for 1.5 h. The completed reaction was concentrated to dryness *in vacuo*, redissolved in DCM (15 mL) and washed with sat. $NaHCO_3$ (aq., 15 mL), followed by NaOH (aq., 0.1 M, 15 mL). The organic phase was concentrated *in vacuo* and purified (Flash column chromatography, Isolera, SNAP 10 g, 2-5% MeOH/DCM), the solvent removed and the compound dried under high vacuum to give compound **3** as a colourless viscous oil (0.37 g, 1.4 mmole, 70%). **$C_{14}H_{21}NO_4$ M_W: 267.32**

R_f (15% MeOH/DCM + TEA) = 0.79

^{1}H NMR (400 MHz, $CDCl_3$) δ 5.85 (s, 1H, NH), 4.06 – 3.93 (m, 4H, $C\underline{H}_2O$, CH_2NH), 2.69 (t, *J* = 6.4 Hz, 2H, $C\underline{H}_2COO$), 2.49 (t, *J* = 6.4 Hz, 2H, $C\underline{H}_2CH_2COO$), 2.29 – 2.09 (m, 4H, HC, CH_3), 1.70 – 1.52 (m, 4H, $C\underline{H}_2CH_2C\underline{H}_2CH_2O$), 1.44-1.36 (m, 2H, $C\underline{H}_2CH_2CH_2O$).

^{13}C NMR (75.5 MHz, CDCl$_3$) δ 206.90 (C), 172.78 (C), 172.45 (C), 79.66 (C), 71.46 (CH), 64.42 (CH$_2$), 37.96 (CH$_2$), 36.19 (CH$_2$), 29.89 (CH$_3$), 29.11 (CH$_2$), 28.25 (CH$_2$), 27.97 (CH$_2$), 25.53 (CH$_2$), 25.04 (CH$_2$).

LRMS: [ES$^+$MS, MeCN] m/z (%): 290 ([M+Na]$^+$, 100 %)

Levulinyl dT precursor **5**

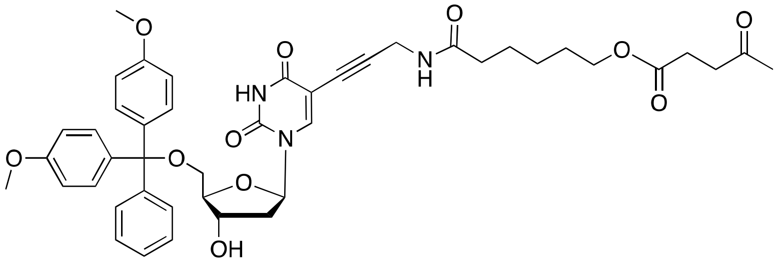

Copper (I) iodide (0.05 g, 0.25 eq., 0.3 mmole), Et$_3$N (0.5 mL), 5'-*O*-(4,4'-dimethoxytrityl)-2'-deoxy-5-iodoruridine, **4** (0.32 g, 1.2 mmole, 1.2 eq.), compound **3** (0.66 g, 1.0 mmole, 1.0 eq.) were dissolved in anhydrous DMF (5 mL) and stirred under an inert Argon atmosphere for 15 minutes before the addition of Pd^0(PPh$_3$)$_4$ (0.12 g, 0.1 mmole, 0.1 eq.). The reaction was complete at 1.5 h then concentrated to dry *in vacuo*. The crude mixture was redissolved in EtOAc (50 mL), washed with EDTA (pH 8, adjust pH using NaOH (aq. 1 M), 20 mL x 3) and brine (20 mL). The organic phase was dried over sodium sulphate, filtered and the filtrate was concentrated *in vacuo* to dryness and purified (Flash column chromatography, Isolera, SNAP 10 g, 80-100% EtOAc/petroleum ether (40-60), 0-10% MeOH/EtOAc + 0.1% pyridine) to give compound **5** as a yellow foam after concentration *in vacuo* and drying under high vacuum for 15 hours (0.47 g, 0.6 mmole, 59%). **$C_{44}H_{49}N_3O_{11}$ M$_W$: 795.87**

^{1}H NMR (400 MHz, DMSO-*d*$_6$) δ 11.65 (s, 1H, NH3), 8.16 (t, *J* = 5.3 Hz, 1H, NHC=O), 7.90 (s, 1H, H6), 7.46 – 7.37 (m, 2H, DMT ArH), 7.36 – 7.18 (m, 7H, DMT ArH), 6.94 – 6.85 (m, 4H, DMT ArH), 6.10 (t, *J* = 6.7 Hz, 1H, H1'), 5.32 (d, *J* = 4.5 Hz, 1H, 3'OH), 4.29-4.25 (m, 4.0 Hz, 1H, H3'), 3.97-3.89 (m, 5H, CH2OCO, H4', C$\underline{H}_2$NH), 3.74 (s, 6H, OMe), 3.26 (dd, *J* = 10.5, 5.4 Hz, 1H, H5'), 3.08 (dd, *J* = 10.6, 3.1 Hz, 1H, H5'), 2.69 (t, *J* = 6.5 Hz, 2H, C$\underline{H}_2$C=OCH$_3$), 2.43 (t, *J* = 6.5 Hz, 2H, C$\underline{H}_2$C=OCH$_3$), 2.33 – 2.13 (m, 2H, H2'), 2.12 – 2.01 (m, 5H, NHCOC$\underline{H}_2$, CH$_3$), 1.58 – 1.42 (m, 4H, C$\underline{H}_2$CH$_2$C$\underline{H}_2$CH$_2$OH), 1.26 (m, 2H, C$\underline{H}_2$CH$_2$CH$_2$OH).

^{13}C NMR (101 MHz, DMSO) δ 206.70 (C), 172.21 (C), 171.54 (C), 161.50 (C), 158.03 (C), 149.27 (C), 144.75 (C), 143.19 (CH), 135.63 (C), 135.16 (C), 129.70 (CH), 129.57 (CH), 127.85 (CH), 127.48 (CH), 126.59 (CH), 113.21 (CH), 98.29 (C), 89.59 (C), 85.80 (CH), 84.95 (CH), 73.95 (C), 70.44 (CH), 63.73 (2CH$_2$), 54.98 (OMe), 39.78 (CH$_2$), 37.38 (CH$_2$), 34.86 (CH$_2$), 29.49 (CH$_3$), 28.47 (CH$_2$), 27.82 (CH$_2$), 27.58 (CH$_2$), 24.99 (CH$_2$), 24.67 (CH$_2$).

LRMS: [ES$^+$MS, MeCN] m/z (%): 818.2 ([M+Na]$^+$, 100 %)

HRMS: [ESI$^+$] m/z: found: 818.3256 (M+Na)$^+$; calcd.: 795.8776 (M)$^+$

Monomer **1**

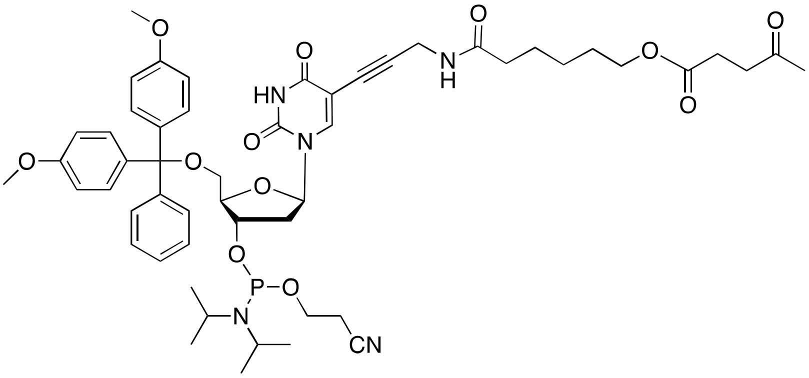

Compound **5** (0.37 g, 0.5 mmole, 1.0 eq.) was dissolved under an inert Argon atmosphere in anhydrous DCM (5 mL) with DIPEA (20 µL, 0.11 mmole, 0.23 eq) and stirred for 15 minutes with molecular sieves (3 Å) to permit the sequestration of water. The simultaneous addition of chloro(diisopropylamino)-β-cyanoethoxyphosphine (130 µL, 0.6 mmole, 1.2 eq.) and DIPEA (170 µL, 0.98 mmole, 1.9 eq.) followed. The reaction was determined complete at 3.5 hours after the further addition of chloro(diisopropylamino)-β-cyanoethoxyphosphine (10 µL, 0.05 mmole, 0.1 eq.). Reaction was concentrated *in vacuo*, diluted with anhydrous DCM (20 mL) and washed with degassed, sat. KCl (aq.), dried over anhydrous Na_2SO_4, filtered then concentrated *in vacuo*. The concentrate was immediately purified by column chromatography (EtOAc (degassed) + 0.1% anhydrous pyridine). Pure fractions were combined and the solvent evaporated *in vacuo* and further dried under high vacuum for 3 hours. The concentrated monomer was redissolved in MeCN (10 mL) and filtered through syringe filtered disc (Acrodisc® PSF Syringe Filters, Pall® Life Sciences). The MeCN was removed *in vacuo* and the monomer dried under high vacuum for 18 hours. The pure monomer, **1**, was obtained as a colourless foam (0.36 g, 0.4 mmole, 74%). **$C_{53}H_{66}N_5O_{12}P$ M_W: 996.09**

^{31}P NMR (121 MHz, Acetonitrile-*d*$_3$) δ 149.31, 149.38.

LRMS: [ES$^+$MS, MeCN] m/z (%): 1018.2 ([M+Na]$^+$, 100 %)

Oligonucleotide synthesis

General procedure

Unmodified oligonucleotides were synthesized on an Applied Biosystems 394 automated DNA/RNA synthesiser using standard conditions. The phosphoramidite monomers and other reagents were purchased from Link Technologies Ltd (Lanarkshire, UK) and Applied Biosystems Ltd (Paisley, UK). All oligonucleotides were synthesised on a standard 1.0 µmole DNA phosphoramidite cycle using the following steps: acid catalysed detritylation, coupling, capping and iodine oxidation. Coupling time for the standard DNA monomers was 40 seconds and the modified monomers (5'-hexynyl phosphoramidite, TFA/MMTr-aminohexyl, HEG and dithiol, octadiynyl-dU and monomer **1** phosphoramidite) was 10 minutes. Cleavage from the solid support was achieved with 35% aqueous ammonia on the synthesiser for 1

hour and further heating at 55 ˚C for 5 hours to remove exocyclic amino protecting groups. Note: in some cases the conditions were modified as described below.

The synthesis of capture probe with modified monomer 1

The capture probe was synthesized using standard conditions until hexynol monomer was incorporated. The oligonucleotide column was then removed from the synthesizer, treated with a solution of diethylamine (20% in acetonitrile for 10 minutes at room temperature, washed four times with 2 mL of acetonitrile and dried under argon for 1 minute) to remove the cyanoethyl protecting group. Then the levulinyl-protecting group was removed using a mixture of hydrazine monohydrate (31 µL), acetic acid (0.5 mL) and pyridine (2.0 mL) at room temperature for 12 minutes. The reaction was washed three times with 2 mL pyridine and three times with 2 mL of acetonitrile and dried under argon for 1 minute before returning to the synthesizer. Finally, the linker was added using standard conditions with 10 minutes coupling time and the resulting oligonucleotide was subjected to standard cleavage and deprotection.

General purification

Purification of oligonucleotides was achieved by reversed-phase HPLC using a Gilson system with an 805 manometric module, 811C dynamic mixer, 306 pump and a 118 UV/vis detector. A Phenomenex C8 column (10 µm, 10 mm × 250 mm) was used for separation. The following protocols were used: run time 20 minutes, flow rate 4 mL/minute, binary gradient: time in minute. (% buffer B); 0 (0); 3 (0); 3.5 (5); 15 (40); 16 (100); 17 (100); 17.5 (0); 20 (0). Buffer A: 0.1 M triethylammonium acetate (TEAA) in water, pH 7.0, buffer B: 0.1 M TEAA in water/acetonitrile (1:1), pH 7.0. Elution of oligonucleotides was monitored by UV absorption. Oligonucleotides were then desalted using NAP 25 and NAP 10 Sephadex columns (G 25, GE-Healthcare), aliquoted into Eppendorf tubes and stored at -20 ˚C. Note: in some cases the conditions were modified i.e., the type of column, buffer and % of buffer B as described below.

Table S1. DNA sequences used in this study

**Sample ID**		**Sequence (5′-3′)**
**Probes**	**Modification**	
Probe-1	Dithiol linker on the thymine	**K**ATATCATCTTTGGTG**T***TTCCTCATGCTTTA
	5′-dithiol	**SSSHH-**ATATCATCTTTGGTGTTTCCTCATGCTTTA
	3′-dithiol	ATATCATCTTTGGTGTTTCCTCATGCTTTA**-HHSSS**
Probe-2	Dithiol linker on the thymine	**K**CACTGACAGTCAGT**T***TGTGGTAGGATGCT
**Targets**		
Target-1/ complementary to probe 1	Unmodified	TAAAGCATGAGGAAACACCAAAGATGATAT
	5′-TR & 3′-Cy3B	**TR-X**TAAAGCATGAGGAAACACCAAAGATGATAT**-Cy3B**
	5′-TR	**TR- X**TAAAGCATGAGGAAACACCAAAGATGATAT
	3′-Cy3B	**X**TAAAGCATGAGGAAACACCAAAGATGATAT **-Cy3B**
Target-2/ non complementary	5′-TR	**TR- X**GGCTACCAGTCGCAGGTAGTTGGTGATAGTC
Target-3/ long complementary to Probe-1	3′-TR & 5′-Cy3	**Cy3-**<br>TAAAGACGTTGTTAAATATTAATCCTAAAGCATGAGGAAACACCAAAGATGATATCGACACACAAACAGGGCTTAATG**–TR**
Target-4/ long complementary to Probe-2	3′-TR & 3′-Cy3	**Cy3-**<br>TGTAAAGACGGCCAGTGCATTCGAGCATCCTACCACAAAACTCACTGTCAGTGTCTGACACACACAAAATGCACGA<br>**-TR**
Target-5/ non complementary	3′-TR	TAAAGACGTTGTTAAATATTAATCCGGCTACCAGTCGCAGGTAGTTGGTGATAGTCCGACACACAAACAGGGCTTAATG-**TR**

[i]K= hexynol, T* = monomer **1**-dithiol-dithiol-dithiol, S = dithiol monomer, H = hexaethylene glycol, TR = amino linkage + Texas Red®, X = 5-Octadiynyl-dU, Cy3B = modified Cy3B, Cy3 = modified Cy3, underline = target region

a) Probe with 3' dithiol anchor

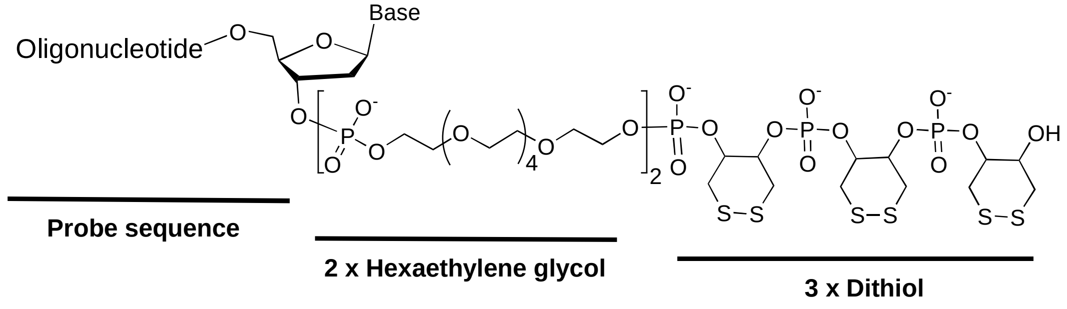

b) Probe with 5' dithiol anchor

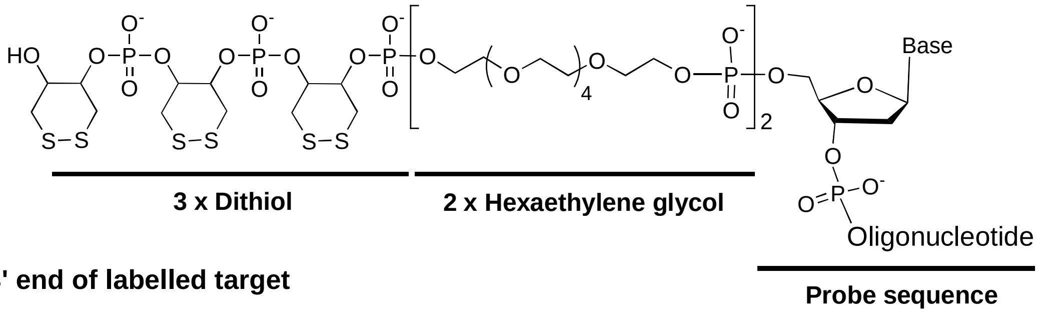

c) Cy3B at 3' end of labelled target

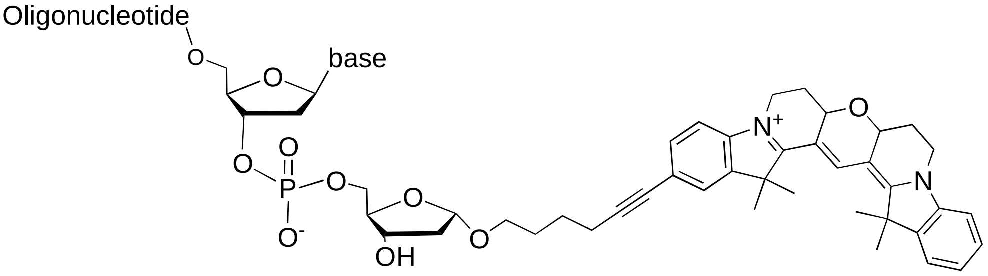

d) Texas red at 5' end of labelled target

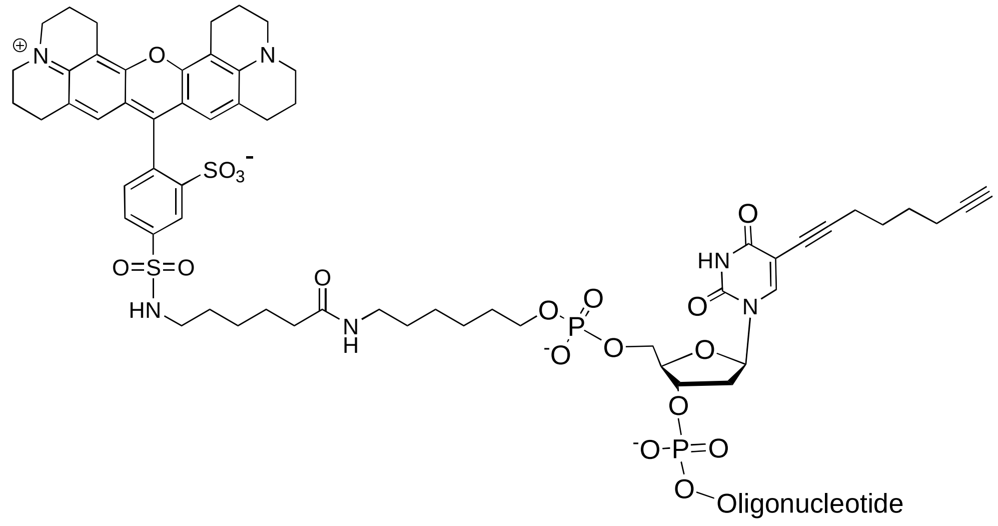

.

e) Texas red at 3' end of labelled target

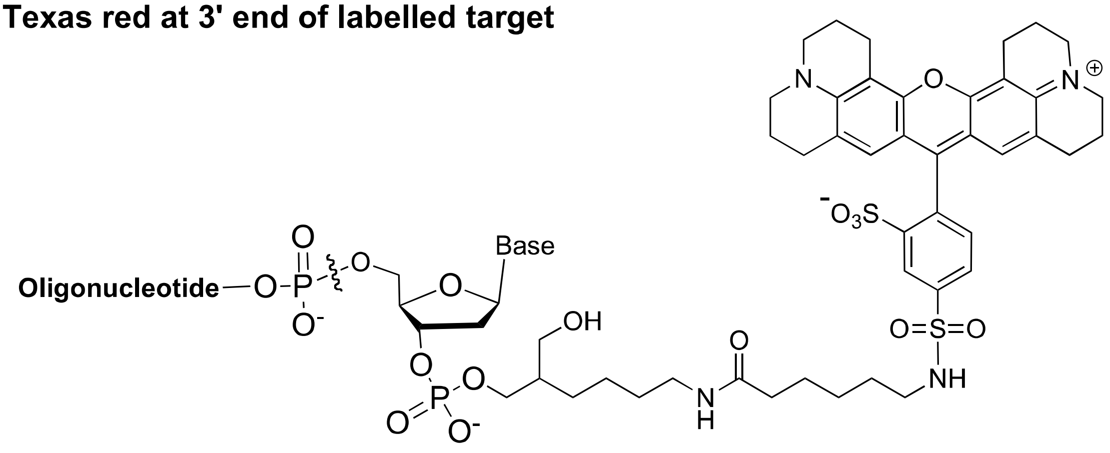

f) Cy3 at 5' end of labelled target

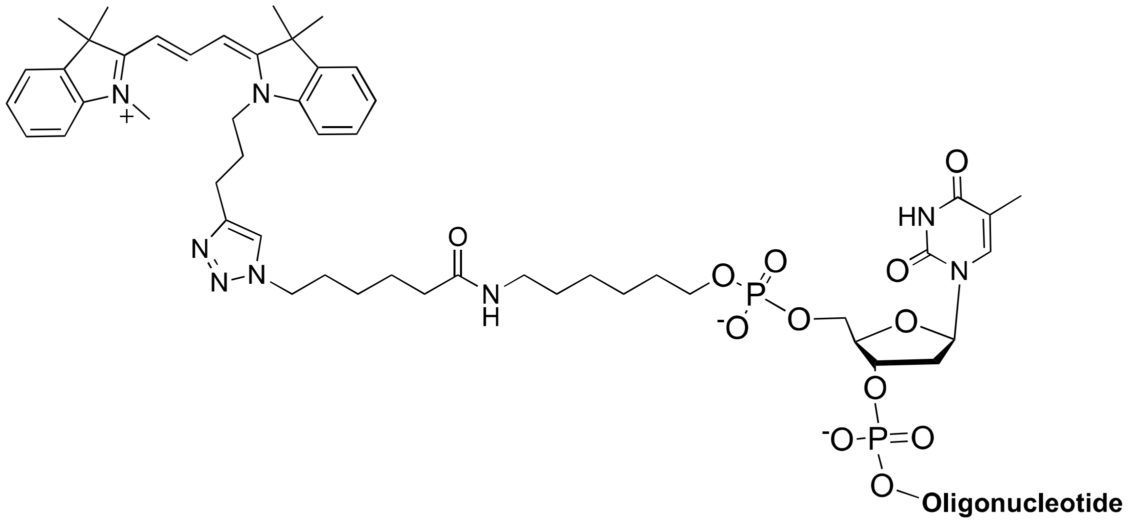

Figure S1 (a) - (b) Structure of dithiol and hexaethylene glycol linker attached at the (a) 3′ and (b) 5' end of the DNA probes. (c) Structure of the Cy3B modification at the 3′ end, (d) the Texas Red modification at the 5′ end, (e) Texas Red modification at the 3′ end, (f). Structure of the Cy3 modification at the 5′ end.

The synthesis of target oligos

The modified targets with Texas Red® and Cy3 were synthesized by post-synthetic labeling with two approaches: amide bond formation for Texas Red® and click chemistry for Cy3 (M. Gerowska, L. M. Hall, J. A. Richardson, M. Shelbourne and T. Brown. *Tetrahedron* **68** (3), 857-864, 2012). Cy3B was attached to a solid support and used with a similar protocol to the standard support (L. M. Hall, M. Gerowska and T. Brown. *Nucleic Acids Res.* **40** (14), 2012).

Target oligos 3 and 4 were synthesized as shown in Figure S2. The oligos were synthesized up to amino link C6 which was protected with MMTr (1 µmole cycle). After removal of the MMTr group, the columns containing oligo bound support were removed from the automated DNA synthesizer and washed with 10 % DEA in acetonitrile (2 mL) over 5 minutes *via*

disposal syringes attached to both ends of the column, washed with acetonitrile (1 mL × 5) and dried under argon for 1 minute. The resin bound oligo was transferred from the column to a plastic vial (2 mL) and a solution of azidohexanoic NHS ester (13 mg, 5 μmole, 5 equiv) in 1% *N*-Methyl morpholine in DMF (80 μL) was added. The reaction was agitated at 450 rpm at 37 ˚C for 4 hours. Each resin bound oligo was transferred to an empty column (Empty Synthesis Columns, TWIST™ Style, Cat. no. 20-0030-00, Glen research), washed with DMF (1 mL × 5), acetonitrile (1 mL × 5), DCM (1 mL × 5) and dried under argon for 1 minute before returning to the automated DNA synthesizer to cleave and deprotect under standard conditions. The crude oligos were purified by RP-HPLC and freeze dried. The pure oligos were labelled with Texas Red® or Cy3.

General protocol for Texas Red® labelling

A solution of Texas Red®-X succinimidyl Ester (Cat.no: T-20175, Life Technologies Ltd, 10 equiv, 500 nmole, 0.4 mg) in DMSO (30 μL) was added to a solution of purified amino-modified oligonucleotide (1 equiv, 50 nmole) in buffer (50 μL, 0.5 M Na_2CO_3/$NaHCO_3$, pH 8.75) and the reaction was agitated at 450 rpm, 55 ˚C, for 5 hours. The reaction mixture was desalted with G-25 gel-filtration column (NAP-25, GE Healthcare, UK) to remove free dyes and the oligonucleotide was then purified by RP-HPLC (as described above but using binary gradient: time in minute. (% buffer B); 0 (0); 3 (0); 3.5 (10); 15 (55); 16 (100); 17 (100); 17.5 (0); 20 (0).).

General protocol for Cy3 labelling

A degassed solution of $CuSO_4$ in water (100 nmol / μL, 10 equiv, 200 nmole, 2 μL) was added to a degassed solution of sodium ascorbate (500 nmol / μL, 100 equiv, 2.0 μmole, 4 μL) and Tris(3-hydroxypropyltriazolylmethyl)amine (70 equiv, 1.4 μmole, 0.60 mg) in water (14 μL) and then the mixture was degassed one more time before being added to a degassed solution of oligos (1 equiv, 40 nmole) and triethylamine (1 μL) in 0.6 M NaCl (20 μL). A solution of degassed Cy3 **11** (10 equiv, 0.40 μmole, 0.20 mg) in DMF (20 μL) was added to the reaction. The resultant was degassed again and agitated at 450 rpm at 30 ˚C for 2 hours (ThermoMixer® C, Eppendorf). The unreacted dye from the reaction was removed using NAP-25 Sephadex columns (GE Healthcare) before purification by reversed-phase HPLC. Conditions: XBridge BEH300 Prep C18 (10 μm, 10 mm × 250 mm), Waters (Peptide Separation Technology). The following protocols were used: run time 20 minutes, flow rate 4 mL/minute, binary gradient: time in minutes. (% buffer B); 0 (0); 3 (0); 3.5 (20); 15 (70); 16 (100); 17 (100); 17.5 (0); 20 (0). Buffer A: 0.1 M ammonium acetate in water, pH 7.0, buffer B: 0.1 M ammonium acetate in water/acetonitrile (3:7), pH 7.0. Elution of oligonucleotides was monitored by UV absorption. Oligonucleotides were then desalted using NAP 10 Sephadex columns (G 25, GE-Healthcare), aliquoted into Eppendorf tubes and stored at -20 ˚C.

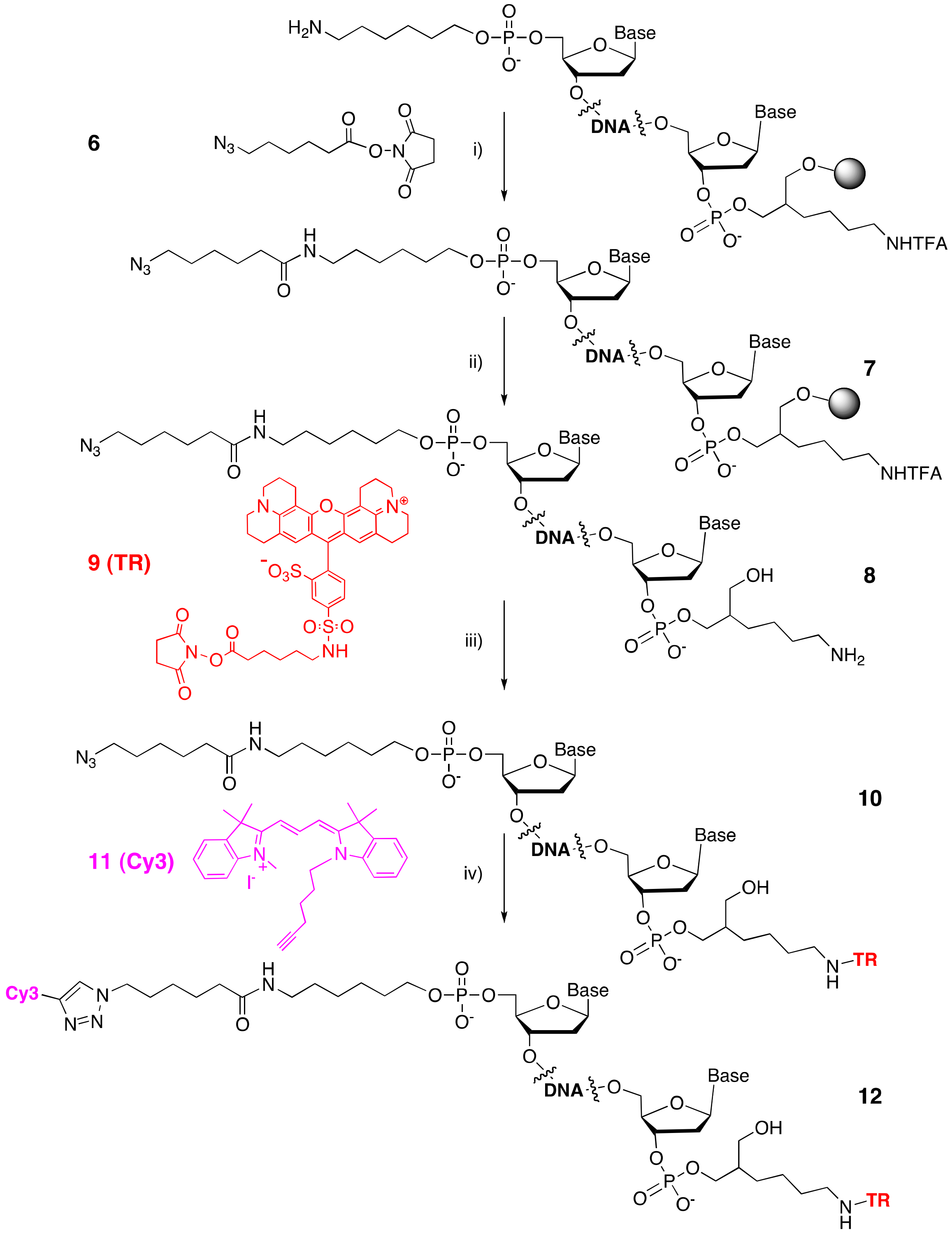

Scheme S1: synthetic pathway to Cy3 and Texas Red® target oligonucleotides i) azide **6** (5 equiv), 4-*N*-methylmorpholine in DMF (1%, 80 μL), oligo on solid support (1 μmole), 37 ˚C, 3 hours; ii) NH_4OH (aq, 35%), rt, 1 and 55 ˚C, 5 h; iii) Texas Red **9** (10 equiv.), DMSO (30 μL), oligo **8** (1 equiv), Sodium bicarbonate buffer (0.5 M, pH 8.75, 50 μL), 55 ˚C, 5 h; iv) oligo **10** (1 equiv), $CuSO_4$ (10 equiv), sodium ascorbate (100 equiv), Tris(3-

hydroxypropyltriazolylmethyl)amine (70 equiv), Cy3 **11** (10 equiv), aq. NaCl: DMF (4:2, v/v, final conc. = 0.2 M), 30 ˚C, 2 h.

Oligonucleotide characterisation

The purity of the oligonucleotides was determined by HPLC-ESI MS analysis by injecting 2 μL of the oligonucleotides (~20-40 μM in water) to a HPLC-mass spectrometry (Bruker Daltronics micrO-TOF mass spectrometer), equipped with a Dionex UltiMate 3000 liquid chromatography system, UV/visible detector, heated column compartment, chilled autosampler (Dionex, Hemel Hempstead, UK) and an Acquity UPLC BEH C18 (1.7 μm, 1 mm × 100 mm column, Waters, Milford, MA, USA). The column temperature was set to 40 ˚C and UV absorbance was measured at 290 nm. A binary phase solvent system was used a gradient of acetonitrile in triethylammonium acetate (TEAA) buffer pH 7 (5-50% buffer B over 15 minutes and then 50-100% buffer B over 4.7 min), with a flow rate of 0.1 mL min^{-1}. Buffers used: Buffer A: 10 mM TEAA, 100 mM HFIP (hexafluoroisopropanol) in water; Buffer B: 20 mM TEAA in acetonitrile. Negative ion ESI data were acquired over the *m*/*z* range 250 – 3500. Data were analysed using Data Analysis™ software v4.0. Raw data were processed/deconvoluted using the DataAnalysis function of the Bruker Daltronics Compass™ 1.3 software package.

Representative HPLC-MS of the capture probes and targets

**Sample ID**		Calc. MS	Found MS
**Probes**	**Modification**		
Probe-1	Dithiol linker on the thymine	10073.58	10074.3799
	5′-dithiol	10449.95	10449.5772
	3′-dithiol	10449.95	10448.8193
Probe-2	Dithiol linker on the thymine	10262.67	10262.4386
**Targets**			
Target-1/ complementary to probe 1	Unmodified	9288.91	9288.6827
	5′-TR & 3′-Cy3B	11258.44	11260.3309
	5′-TR	10563.24	10563.2063
	3′-Cy3B	10378.44	10378.3884
Target-2/ non complementary	5′-TR	10487.04	10487.8310
Target-3/ long complementary to Probe-1	3′-TR & 5′-Cy3	25762.21	25759.2392
Target-4/ long complementary to Probe-2	3′-TR & 3′-Cy3	24946.66	24950.4075
Target-5/ non complementary	3′-TR	25355.34	25356.0643

HPLC-MS of Probe 1: Dithiol linker on the thymine at 290 nm

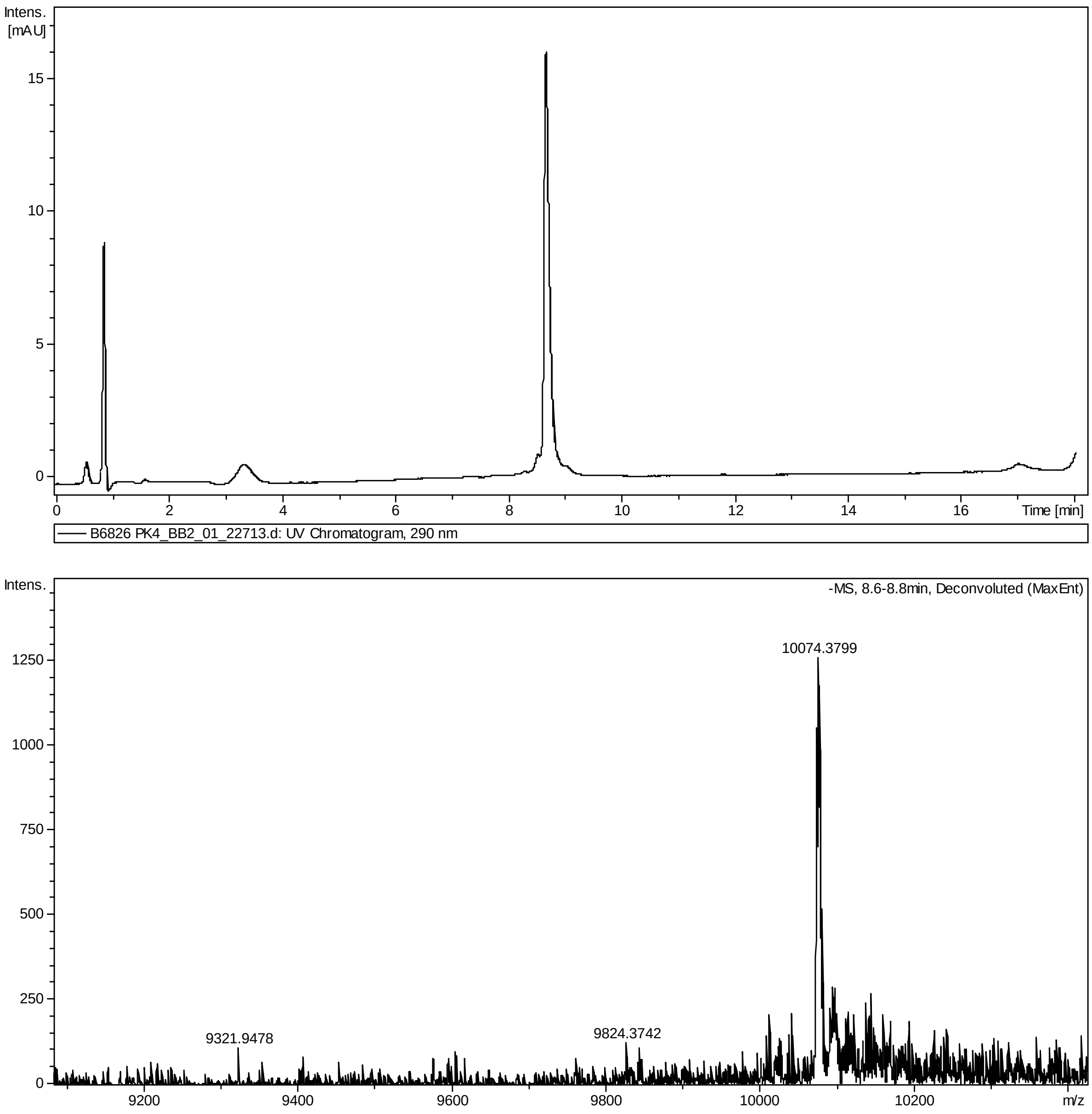

HPLC-MS of Probe 2: Dithiol linker on the thymine at 290 nm

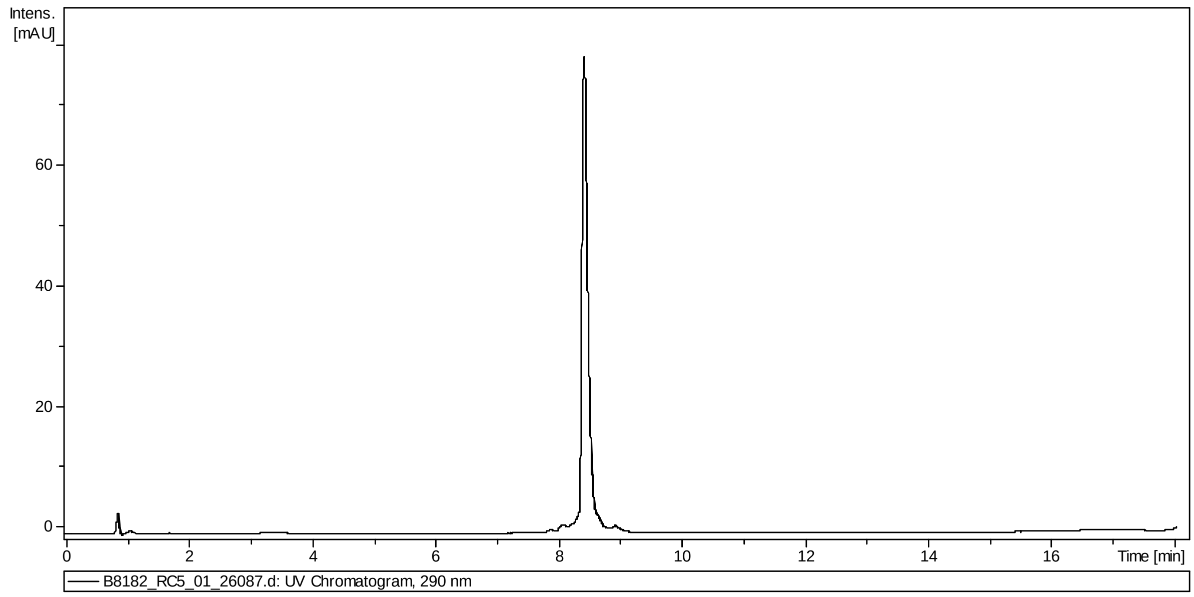

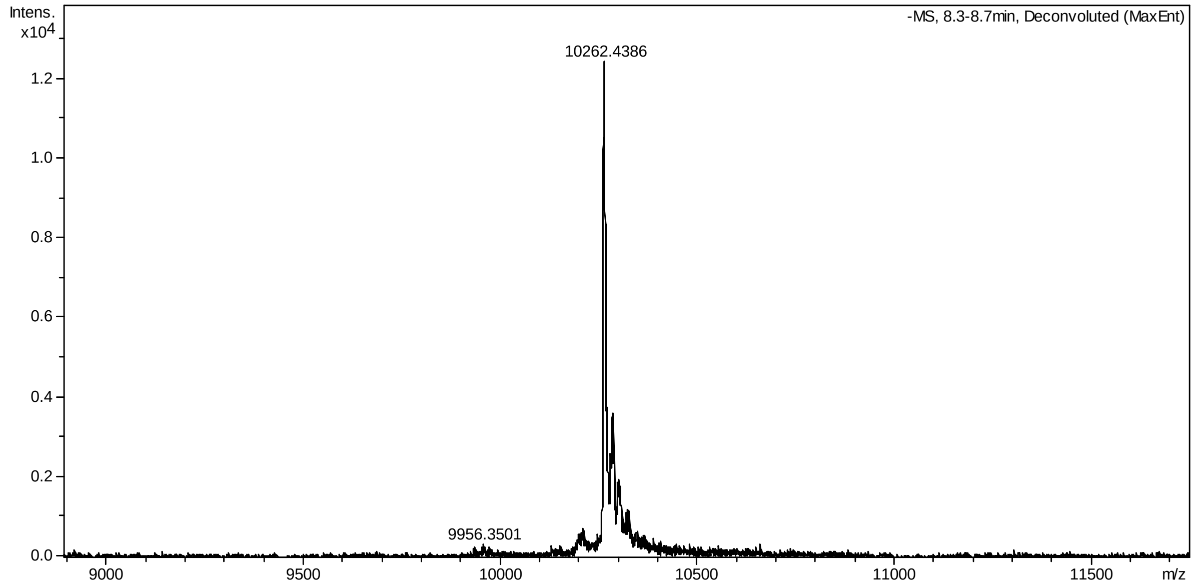

HPLC-MS of target 4 at 290 nm

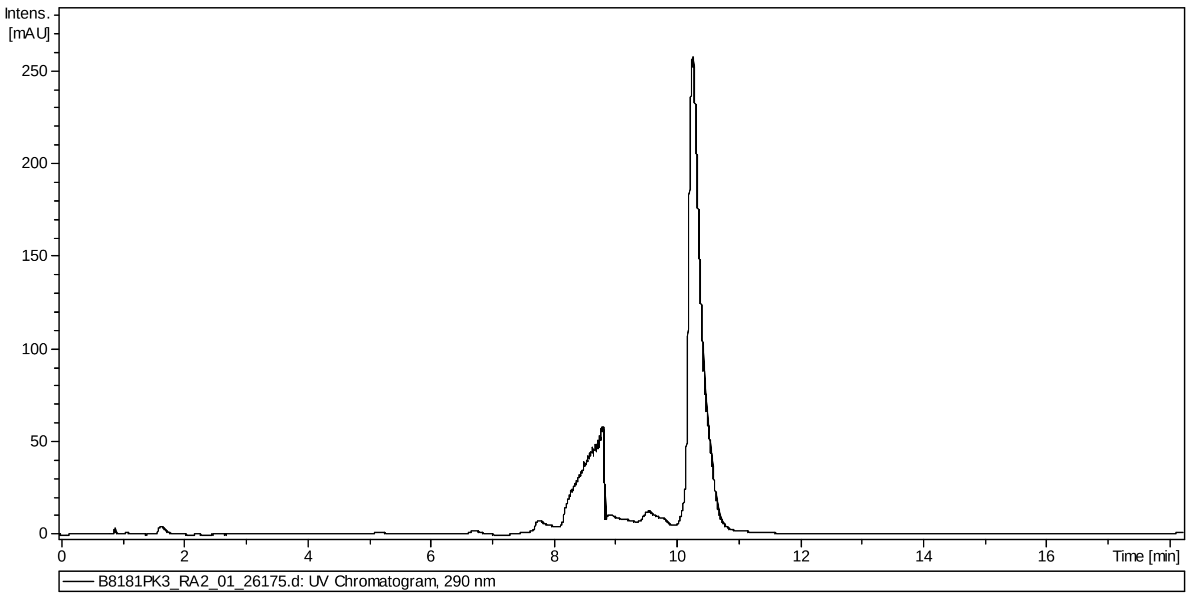

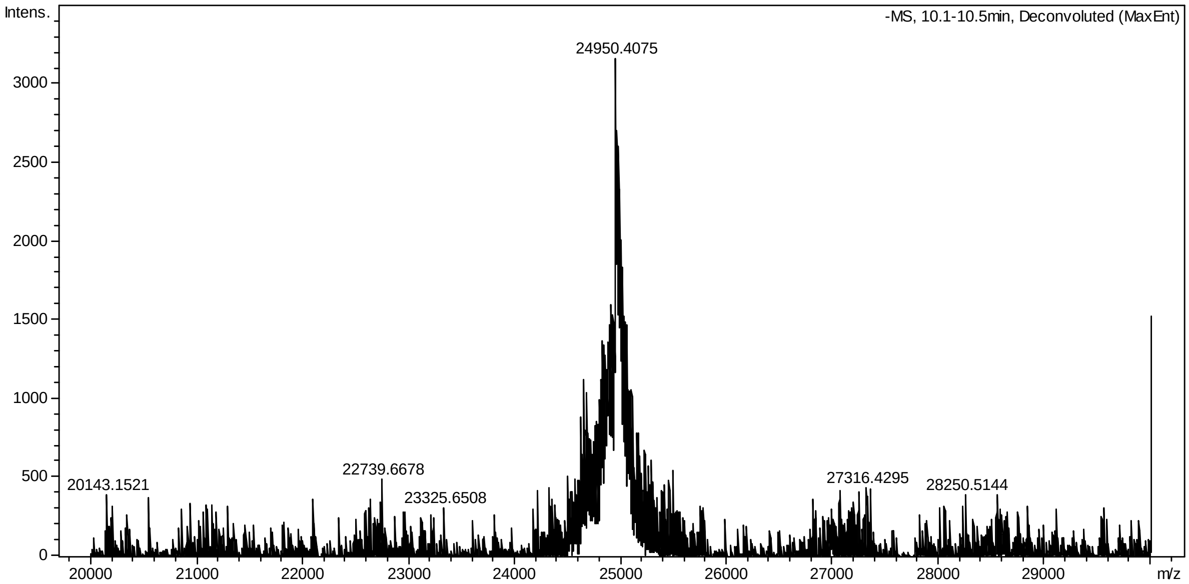

Preparation of Sphere Segment Void (SSV) Substrates

A gold-chrome coated microscope slide was prepared by thermal vapour deposition of a 10 nm chromium adhesion layer followed by approximately 200 nm of gold onto a standard glass microscope slide. A monolayer template of 600 nm of polystyrene spheres (Fisher Scientific as a 1 % wt aqueous suspension) was formed at the surface using a convective assembly method (S. Mahajan, J. Richardson, T. Brown, P.N. Bartlett, *J. Am. Chem. Soc.*, **130** (2008) 15589-15601). Gold was deposited through the template to a height of 480 nm at -0.72 V vs. SCE from commercial gold plating solution (ECF 60, Metalor) containing 100 μL brightener (E3, Metalor) in 20 ml of plating solution. After deposition the polystyrene spheres were removed by immersion in DMF (HPLC grade, Rathburn Chemicals Ltd, Scotland) for thirty minutes, and the substrates were rinsed in deionised water before immediate use.

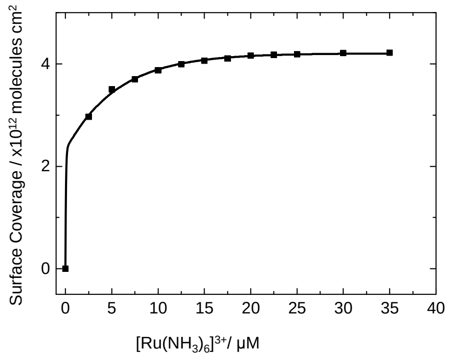

Figure S2. Binding isotherm for $[Ru(NH_3)_6]^{3+}$ with DNA duplex immobilised horizontally on a Au SSV substrate. For this experiment, the duplex was hybridized in solution and then the duplex strand (0.5 µM of dsDNA in 0.05 M Na_2SO_4) was immobilised on the SSV Au surface overnight. The surface was then passivated with mercaptohexanol (1 mM).

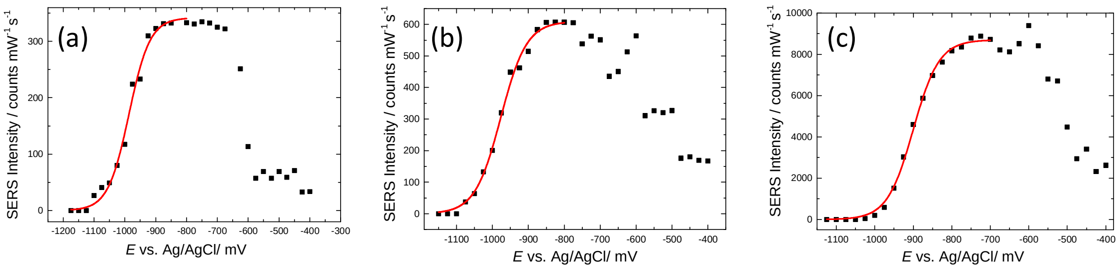

Figure S3. Changes in the peak height of the Texas Red band ~1504 cm^{-1} as a function of applied potential for (a) and (b) horizontally tethered dsDNA immobilised on the surface and (c) vertically tethered DNA. For (a) the DNA was hybridized in solution and then the duplex strand (0.5 µM of dsDNA in 0.05 M Na_2SO_4) was immobilised on the Au SSV surface overnight, (b) the ssDNA probe (0.5 µM of ssDNA in 0.05 M Na_2SO_4) was immobilized on the Au SSV surface at 40 ^{0}C for 6 hours following by hybridization of the complementary DNA at room temperature and (c) the ssDNA probe (1 µM of ssDNA in 1 M NaCl) was immobilized on the Au SSV surface overnight at room temperature following by hybridization with the complementary DNA. All the surfaces were passivated with mercaptohexanol (10 mM).

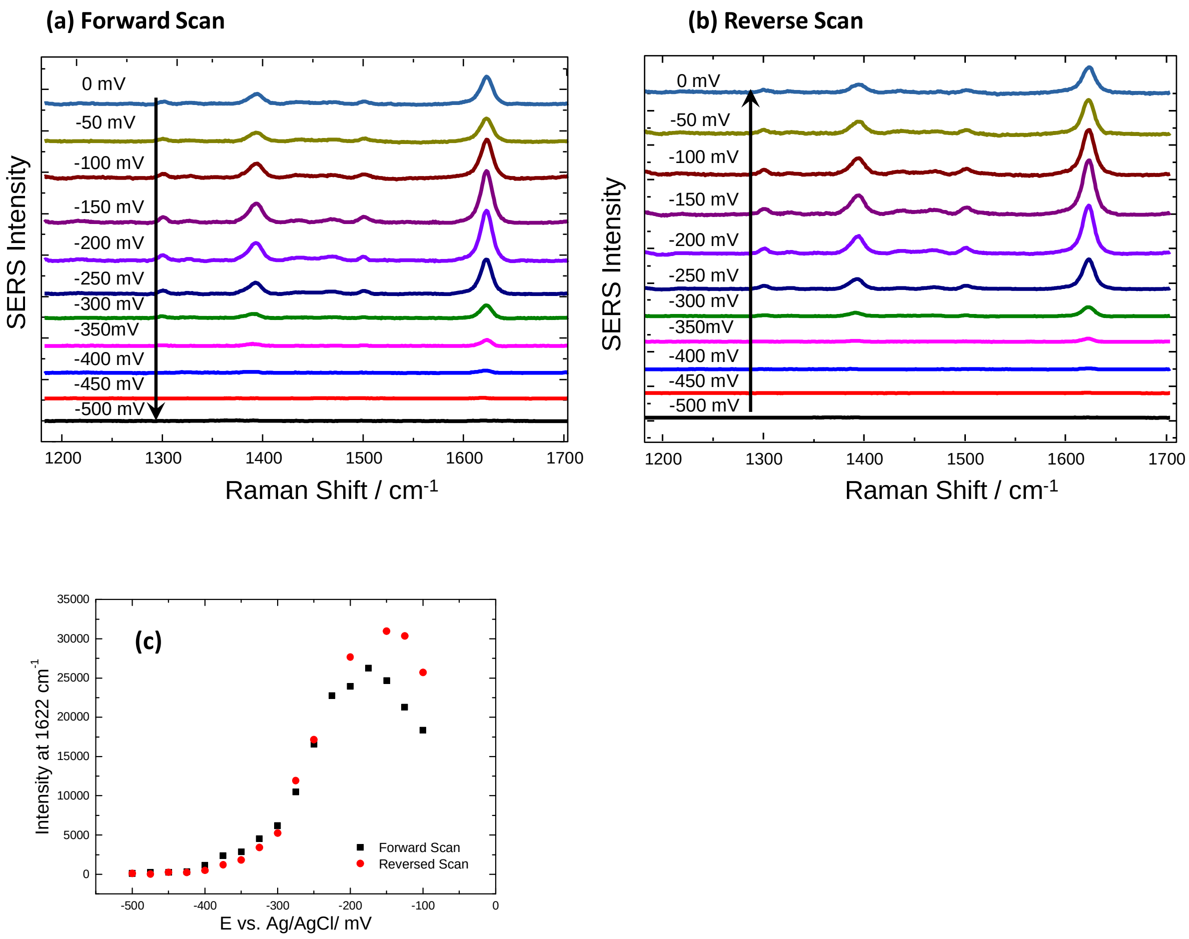

Figure S4. (a) and (b) SERS spectra of methylene blue bound on horizontally tethered dsDNA at applied potentials vs. Ag/AgCl as shown on the figure. For this experiment, the duplex was hybridized in solution and then the duplex strand (0.5 μM of dsDNA in 0.05 M Na_2SO_4) was immobilised on the Au surface overnight. The surface was then passivated with mercaptohexanol (1 mM). The spectra show the reduction and oxidation of methylene blue. The potential was swept at a scan rate of 0.7 mV s^{-1} in 10 mM Tris Buffer (pH 7.2) containing 1 M NaCl. (b) Plot of changes in the absolute signal intensities of the band 1622 cm^{-1} as a function of applied potential for both the anodic and cathodic scans.
